# Supplementary material for: Four New ent-Kaurane Diterpene Glycosides from Isodon henryi
Source: Molecules. 2019 Jul 27;24(15):2736. doi: 10.3390/molecules24152736 (PMC6695894; doi:10.3390/molecules24152736)
Supplement: Supplementary file 1 [file molecules-24-02736-s001.pdf]

SUPPLEMENTARY MATERIAL

Supplementary Materials

Supplementary material relating to this article is available online, alongside Figure S1-S36, S38-43 and S37.

Contents

Figure S1. HR-ESI-MS spectrum of compound **1**.
Figure S2. IR spectrum of compound **1**.
Figure S3. ^{1}H-NMR spectrum of compound **1**.
Figure S4. ^{13}C-NMR spectrum of compound **1**.
Figure S5. DEPT spectrum of compound **1**.
Figure S6. HSQC spectrum of compound **1**.
Figure S7. ^{1}H-^{1}H COSY spectrum of compound **1**.
Figure S8. HMBC spectrum of compound **1**.
Figure S9. NOESY spectrum of compound **1**.
Figure S10. HR-ESI-MS spectrum of compound **2**.
Figure S11. IR spectrum of compound **2**.
Figure S12. ^{1}H-NMR spectrum of compound **2**.
Figure S13. ^{13}C-NMR spectrum of compound **2**.
Figure S14. DEPT spectrum of compound **2**.
Figure S15. HSQC spectrum of compound **2**.
Figure S16. ^{1}H-^{1}H COSY spectrum of compound **2**.
Figure S17. HMBC spectrum of compound **2**.
Figure S18. NOESY spectrum of compound **2**.
Figure S19. HR-ESI-MS spectrum of compound **3**.
Figure S20. IR spectrum of compound **3**.
Figure S21. ^{1}H-NMR spectrum of compound **3**.
Figure S22. ^{13}C-NMR spectrum of compound **3**.
Figure S23. DEPT spectrum of compound **3**.
Figure S24. HSQC spectrum of compound **3**.
Figure S25. ^{1}H-^{1}H COSY spectrum of compound **3**.
Figure S26. HMBC spectrum of compound **3**.
Figure S27. NOESY spectrum of compound **3**.
Figure S28. HR-ESI-MS spectrum of compound **4**.
Figure S29. IR spectrum of compound **4**.
Figure S30. ^{1}H-NMR spectrum of compound **4**.
Figure S31. ^{13}C-NMR spectrum of compound **4**.
Figure S32. DEPT spectrum of compound **4**.
Figure S33. HSQC spectrum of compound **4**.
Figure S34. ^{1}H-^{1}H COSY spectrum of compound **4**.
Figure S35. HMBC spectrum of compound **4**.
Figure S36. NOESY spectrum of compound **4**.
S37. ECD calculation details of compound of **1**.

Figure S38. ^{1}H-NMR spectrum of compound **5**.
Figure S39. ^{13}C-NMR spectrum of compound **5**.
Figure S40. ^{1}H-NMR spectrum of compound **6**.
Figure S41. ^{13}C-NMR spectrum of compound **6**.
Figure S42. ^{1}H-NMR spectrum of compound **7**.
Figure S43. ^{13}C-NMR spectrum of compound **7**.

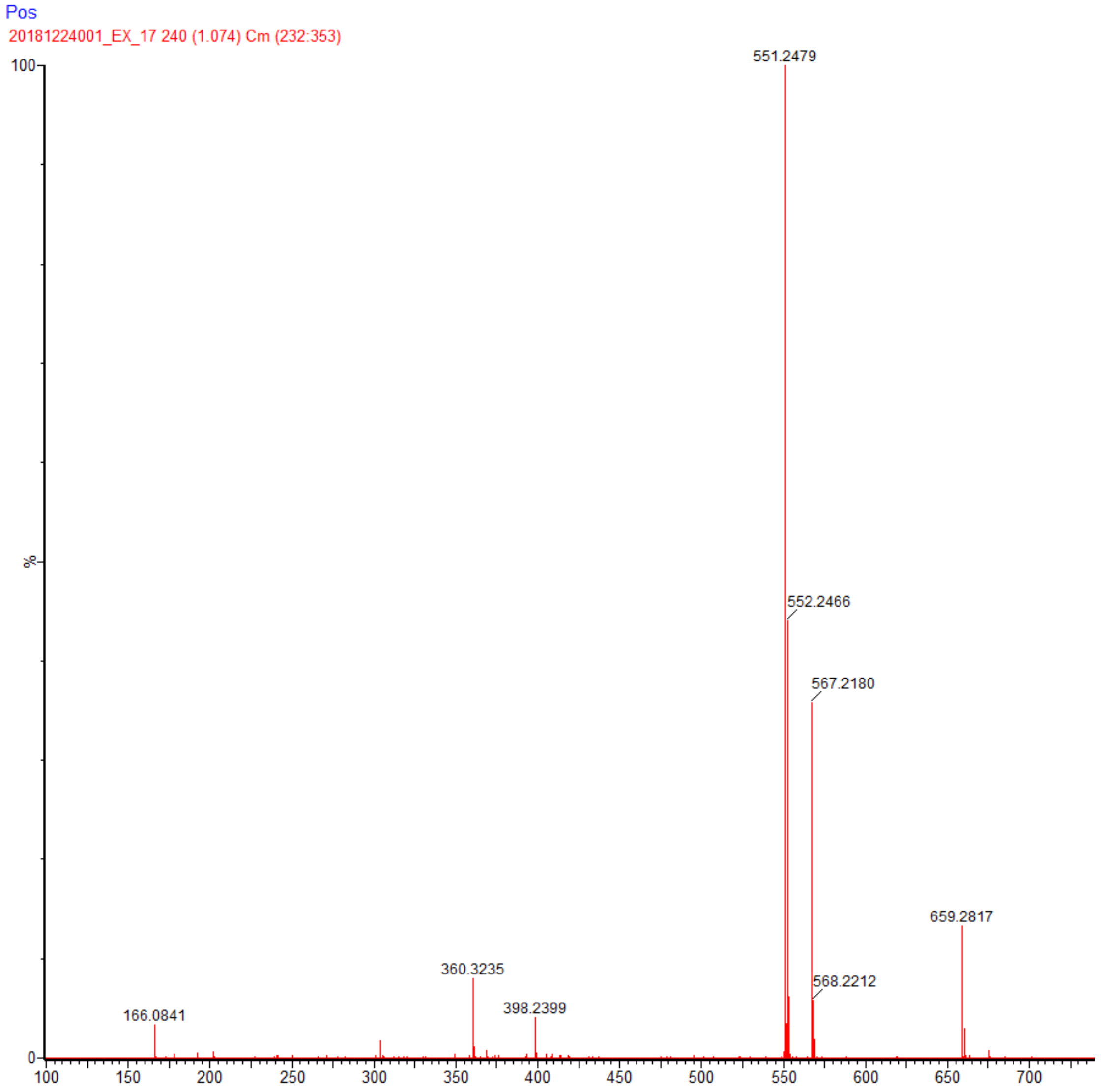

Figure S1. HR-ESI-MS spectrum of compound **1**.

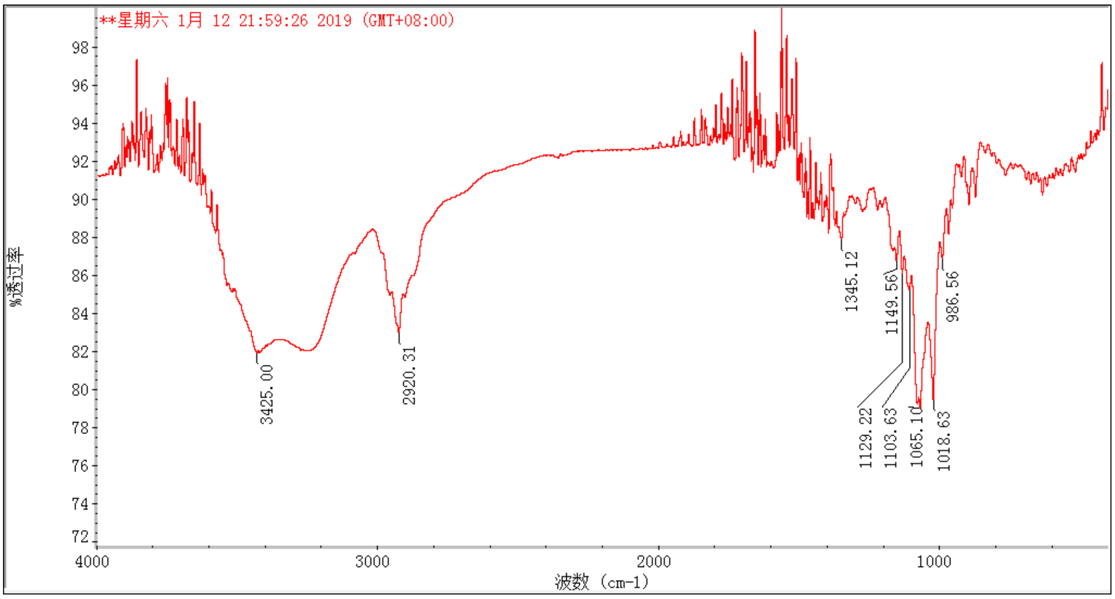

Figure S2. IR spectrum of compound **1**.

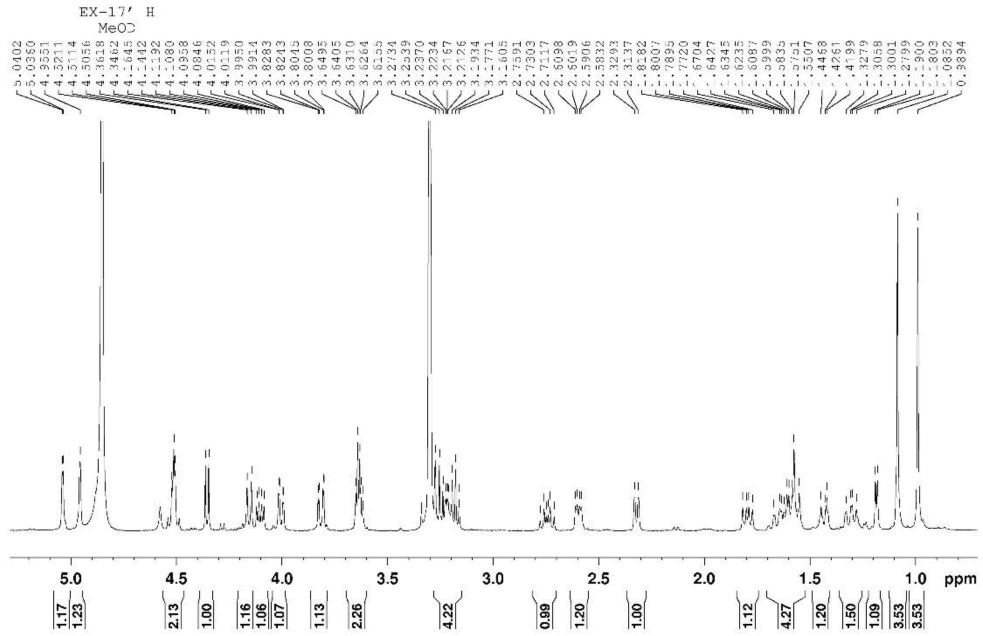

Figure S3. ^{1}H-NMR spectrum of compound **1**.

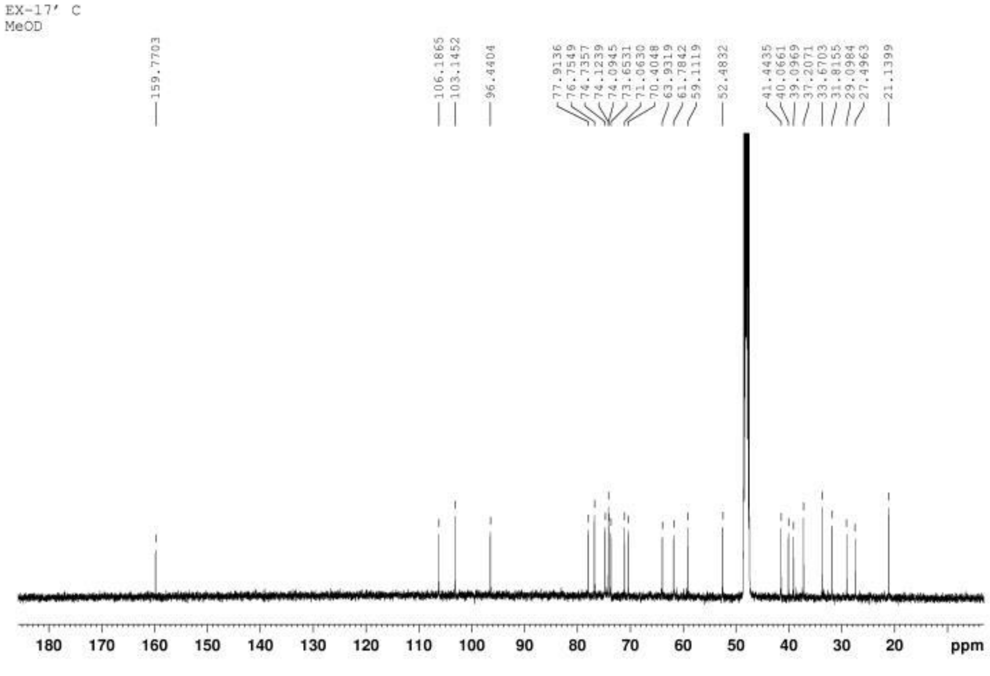

Figure S4. ^{13}C-NMR spectrum of compound **1**.

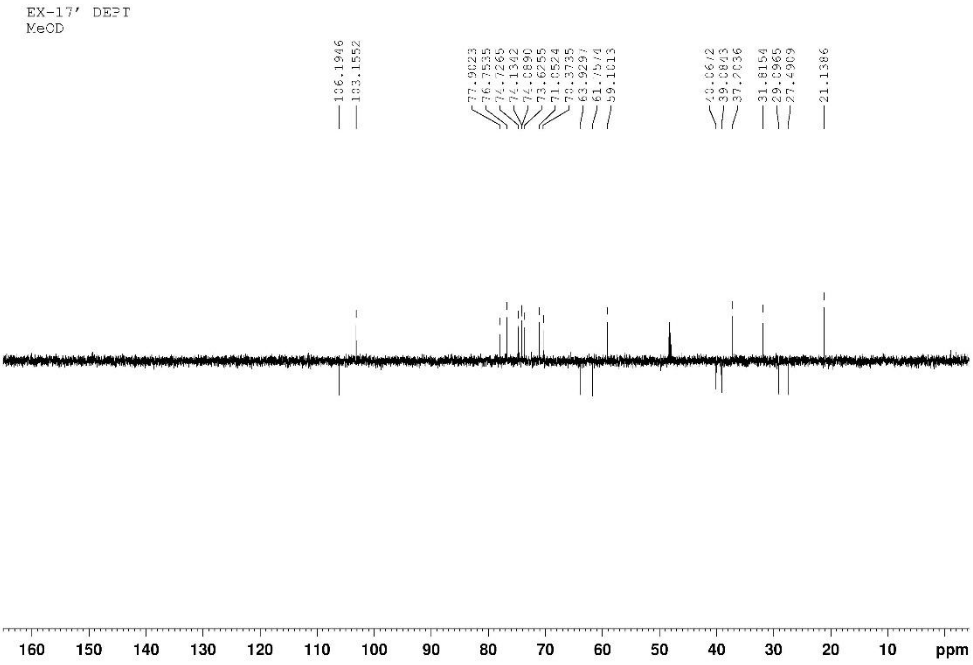

Figure S5. DEPT spectrum of compound **1**.

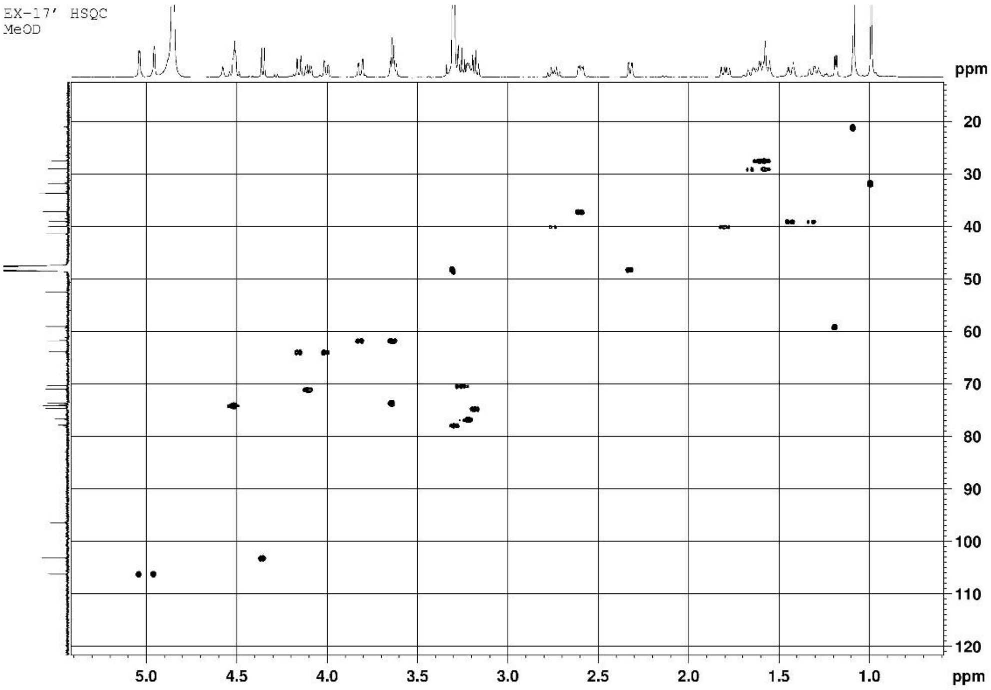

Figure S6. HSQC spectrum of compound **1**.

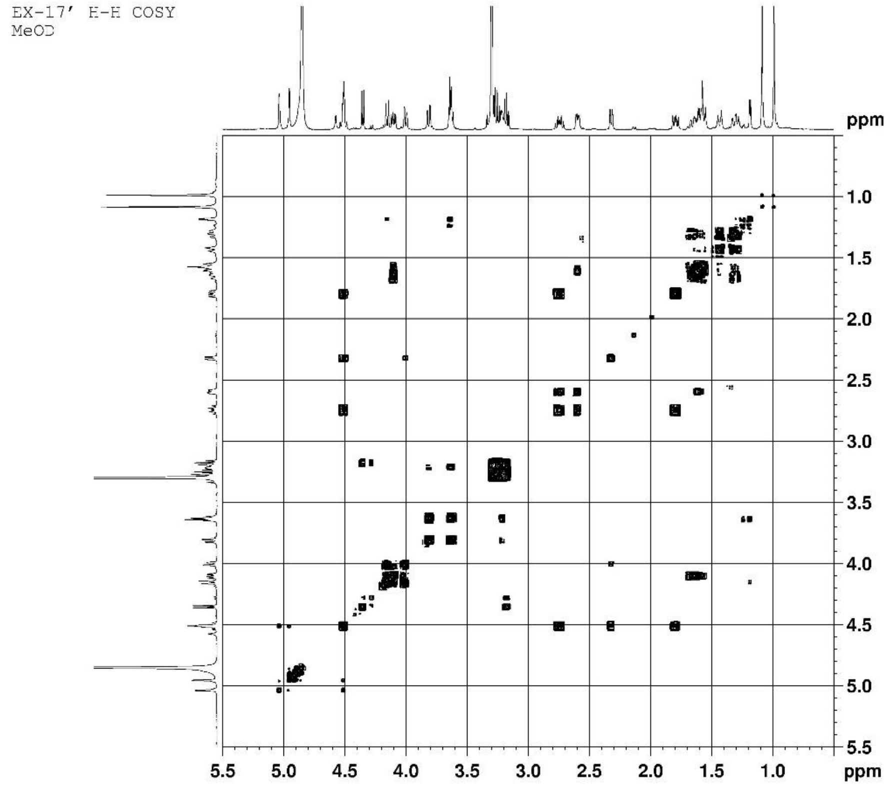

Figure S7. ^{1}H-^{1}H COSY spectrum of compound **1**.

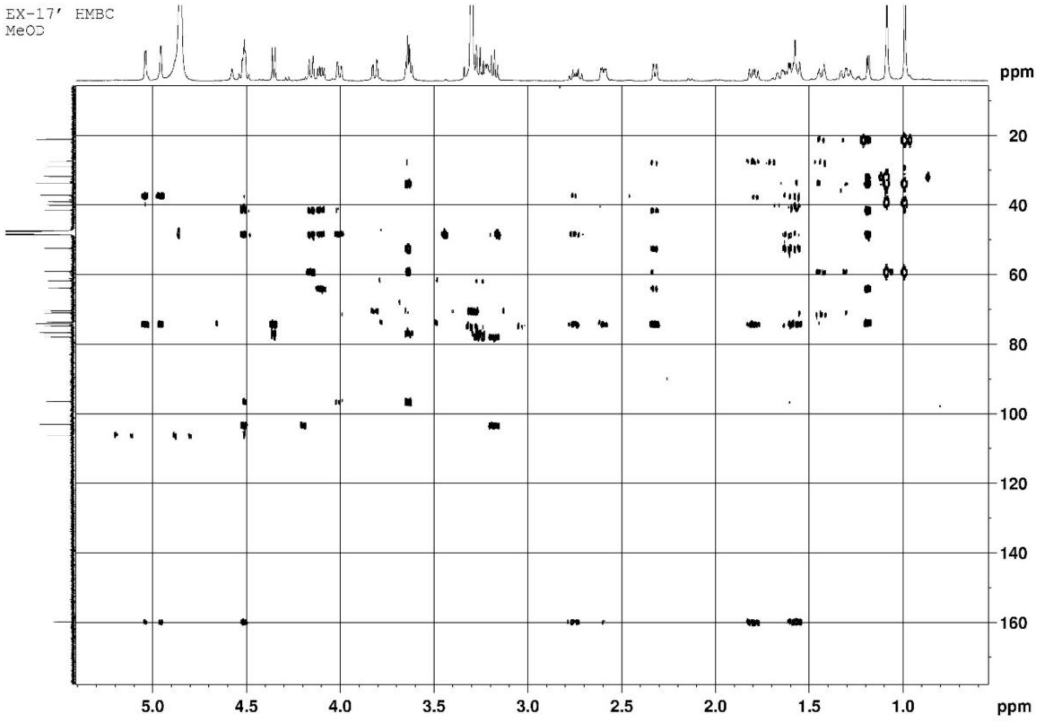

Figure S8. HMBC spectrum of compound **1**.

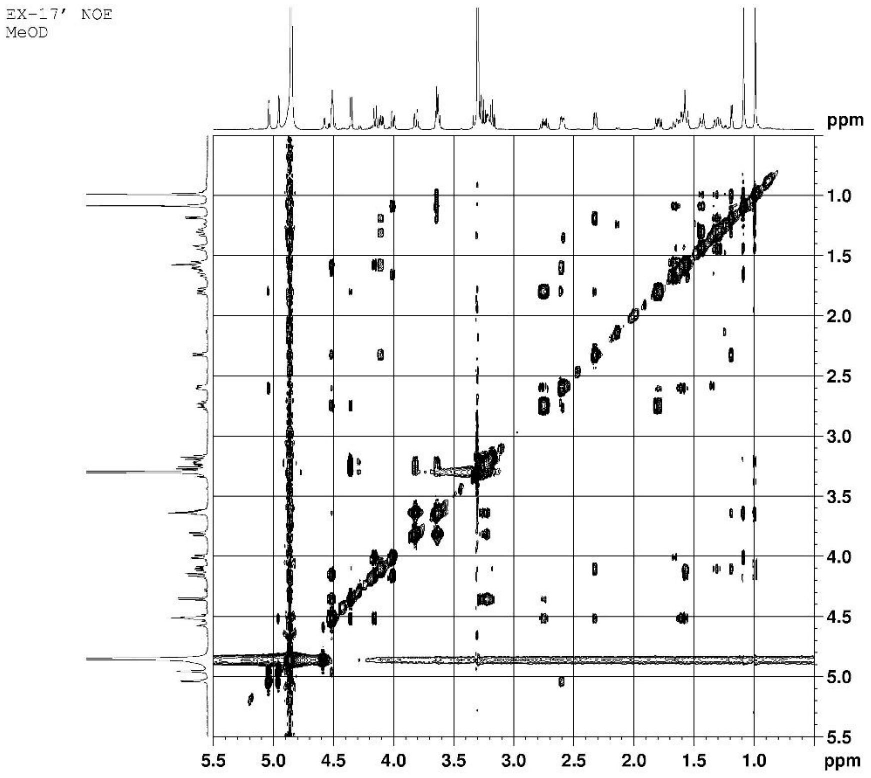

Figure S9. NOESY spectrum of compound **1**.

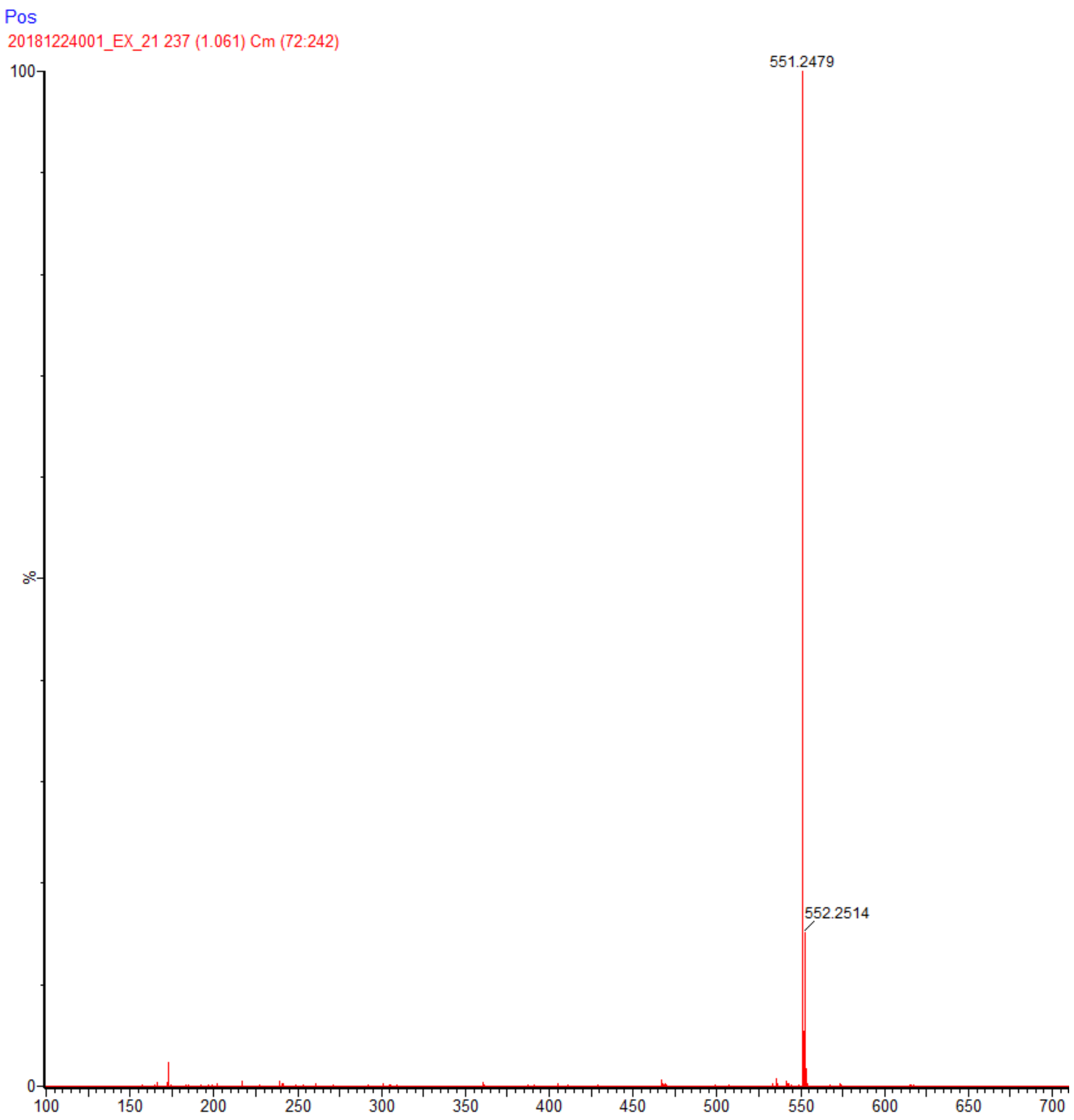

Figure S10. HR-ESI-MS spectrum of compound **2**.

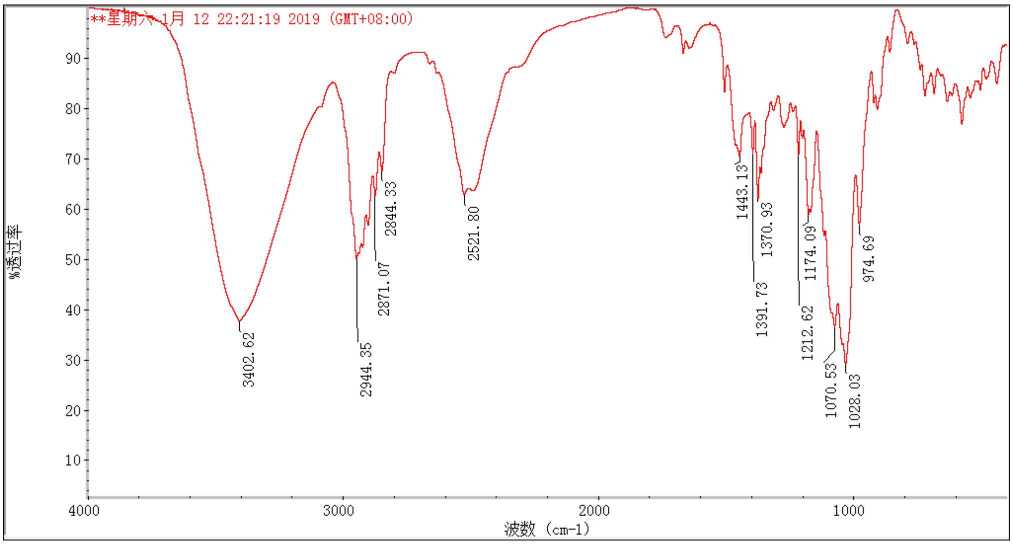

Figure S11. IR spectrum of compound **2**.

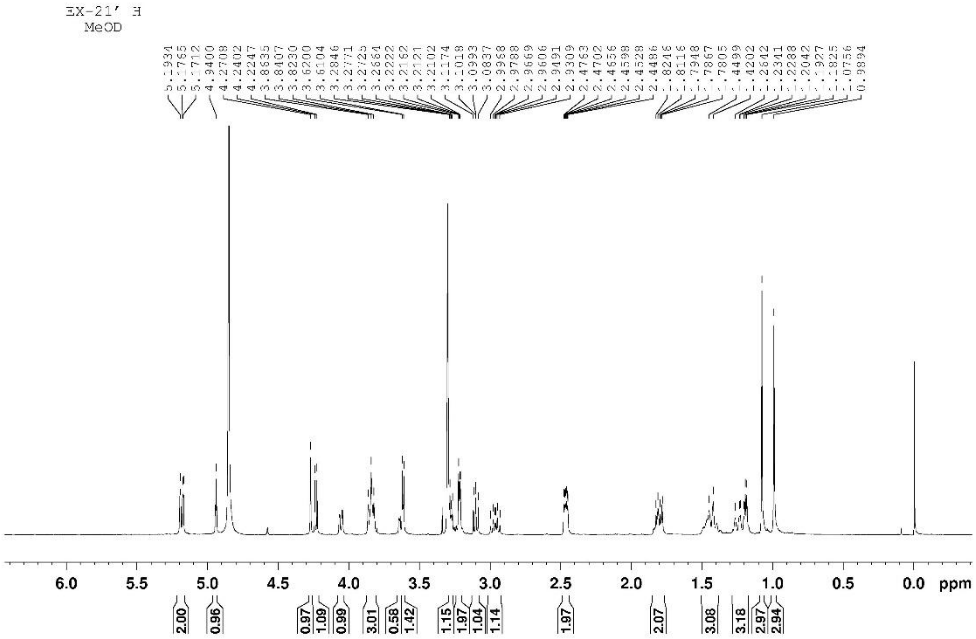

Figure S12. ^{1}H-NMR spectrum of compound **2**.

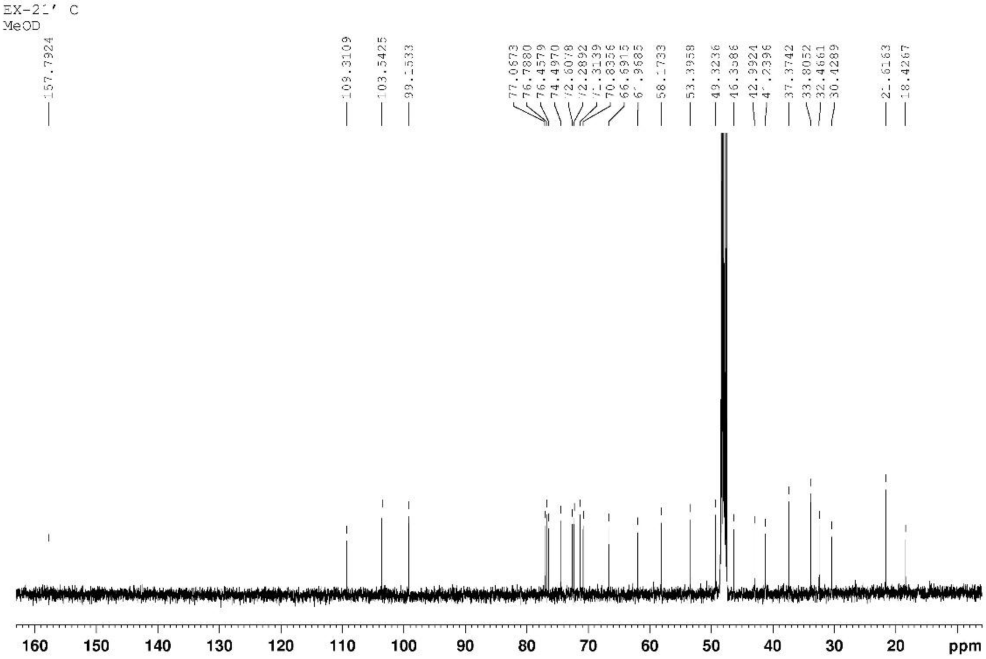

Figure S13. ^{13}C-NMR spectrum of compound **2**.

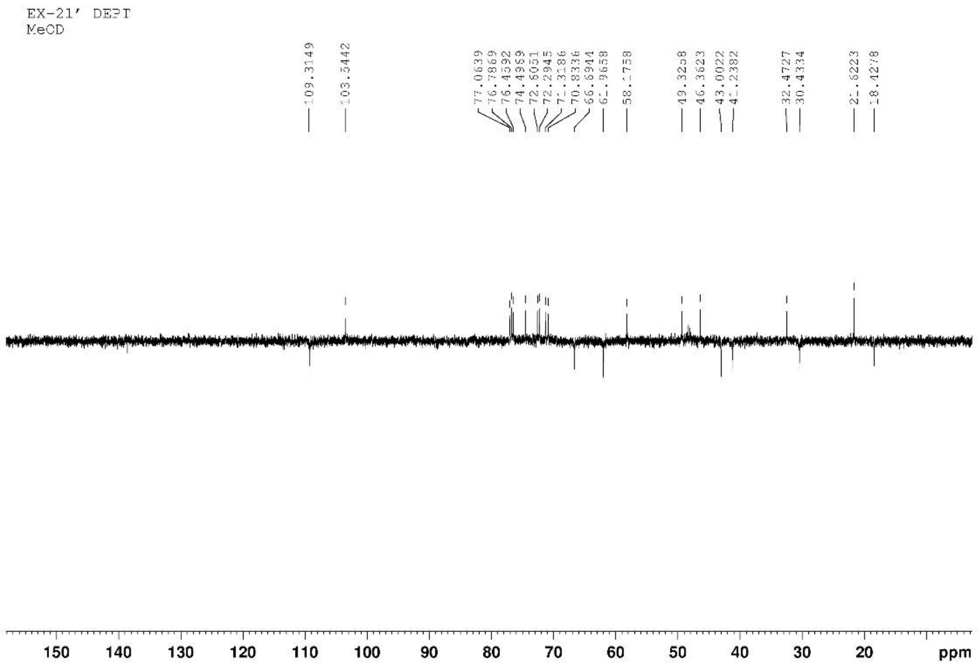

Figure S14. DEPT spectrum of compound **2**.

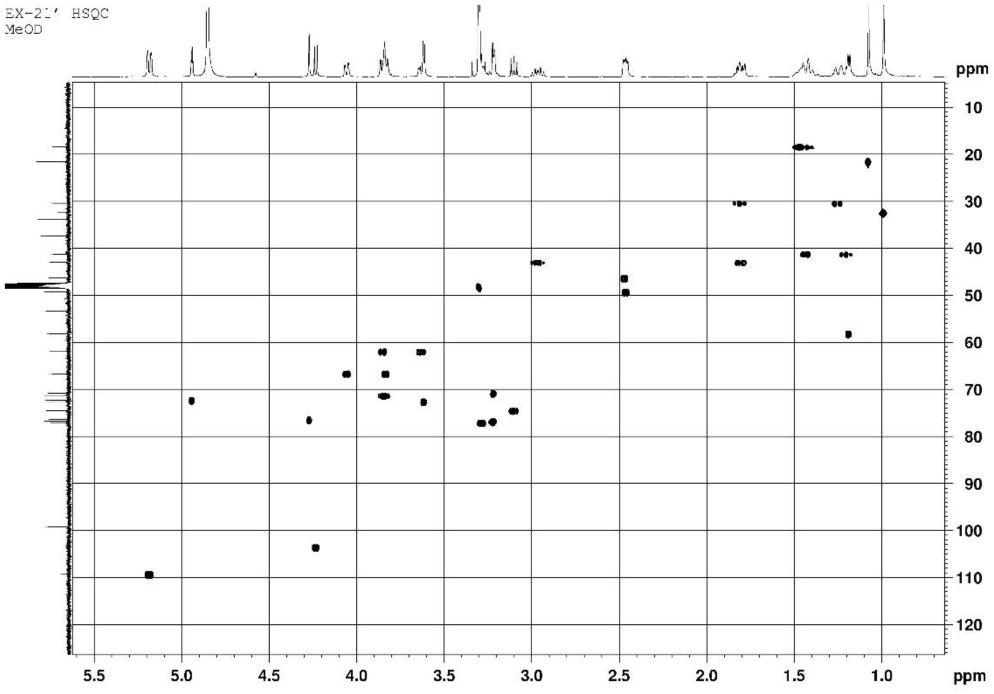

Figure S15. HSQC spectrum of compound **2**.

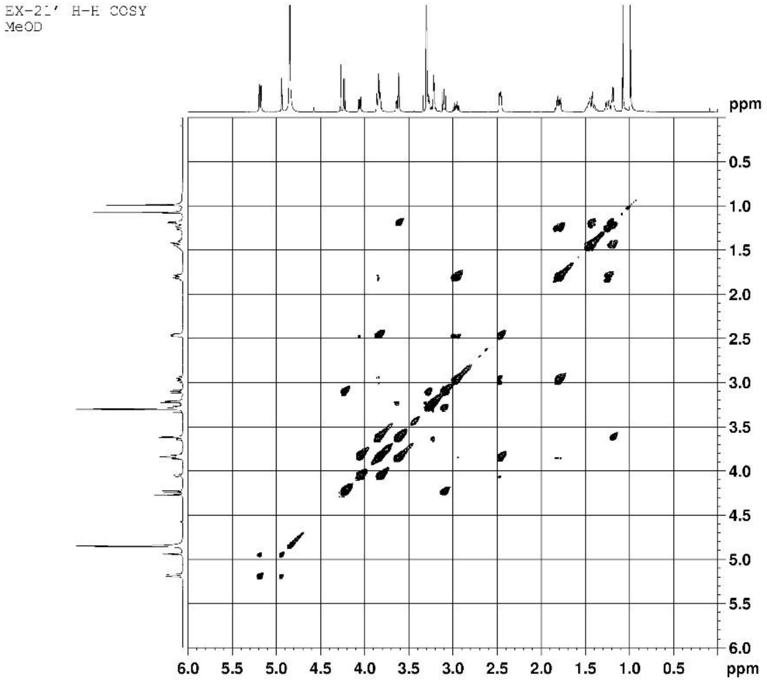

Figure S16. ^{1}H-^{1}H COSY spectrum of compound **2**.

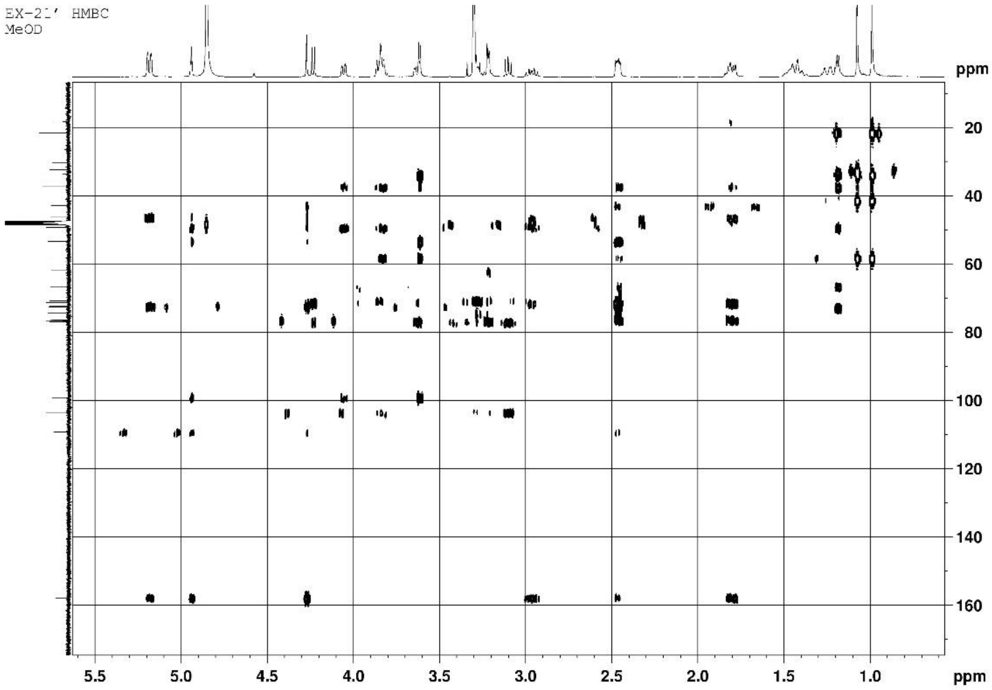

Figure S17. HMBC spectrum of compound **2**.

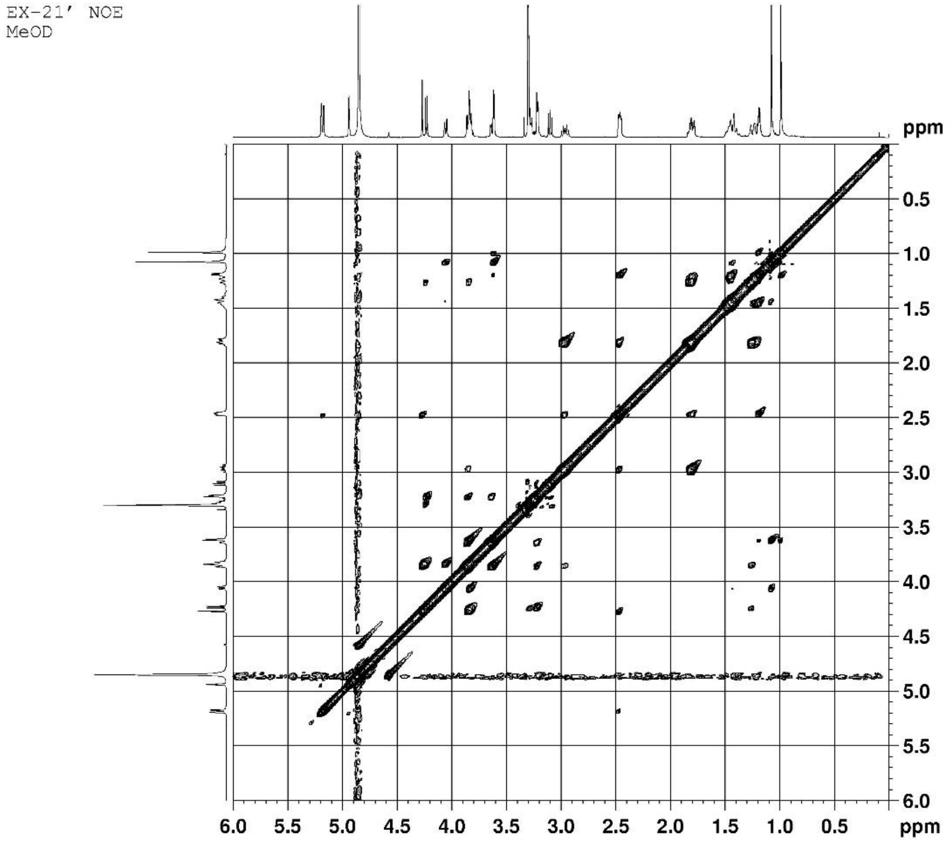

Figure S18. NOESY spectrum of compound **2**.

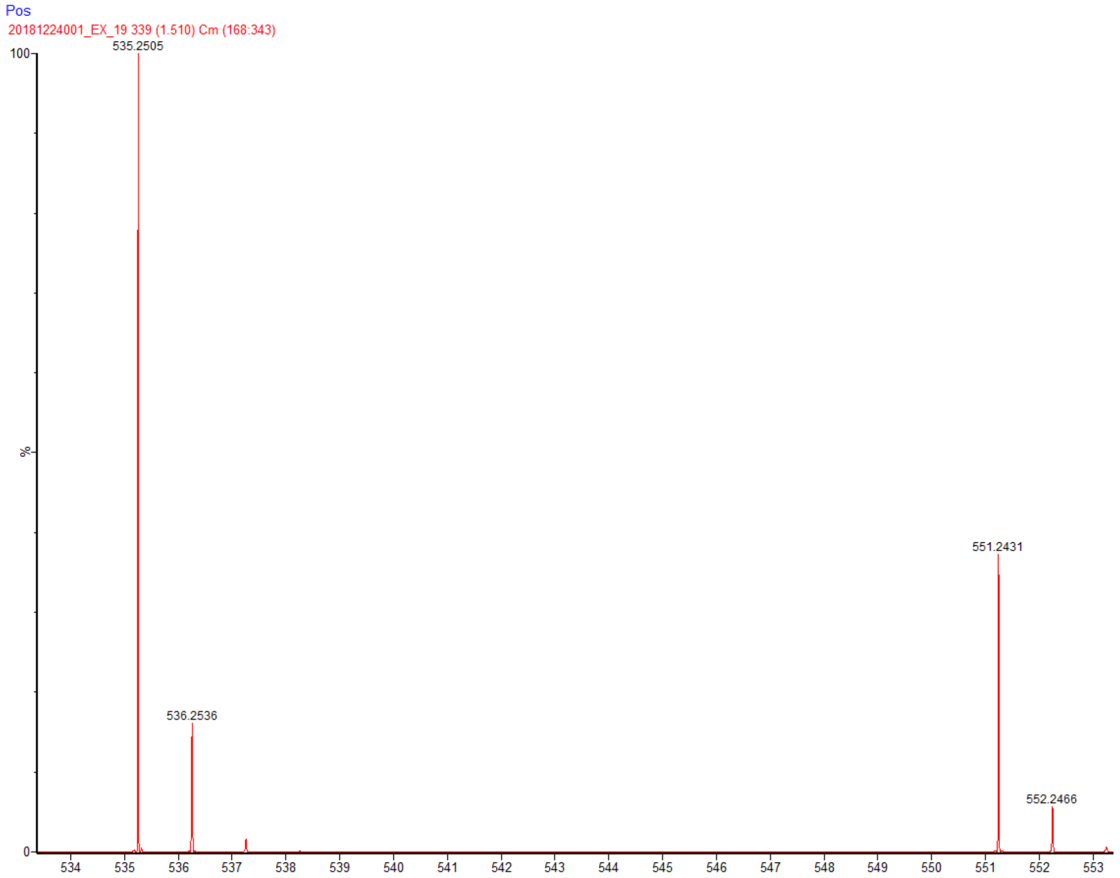

Figure S19. HR-ESI-MS spectrum of compound **3**.

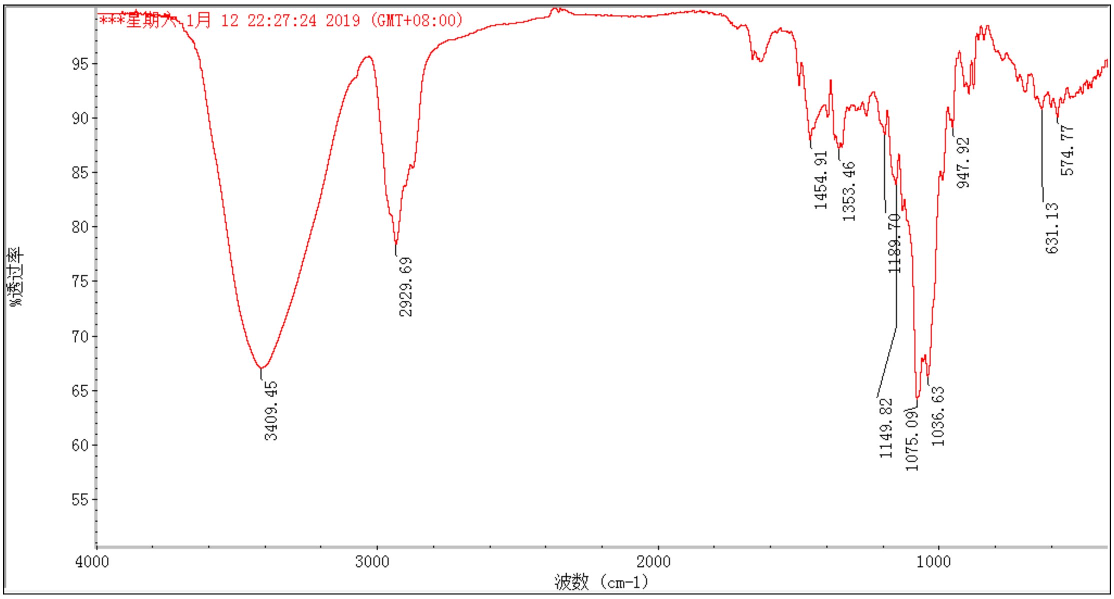

Figure S20. IR spectrum of compound **3**.

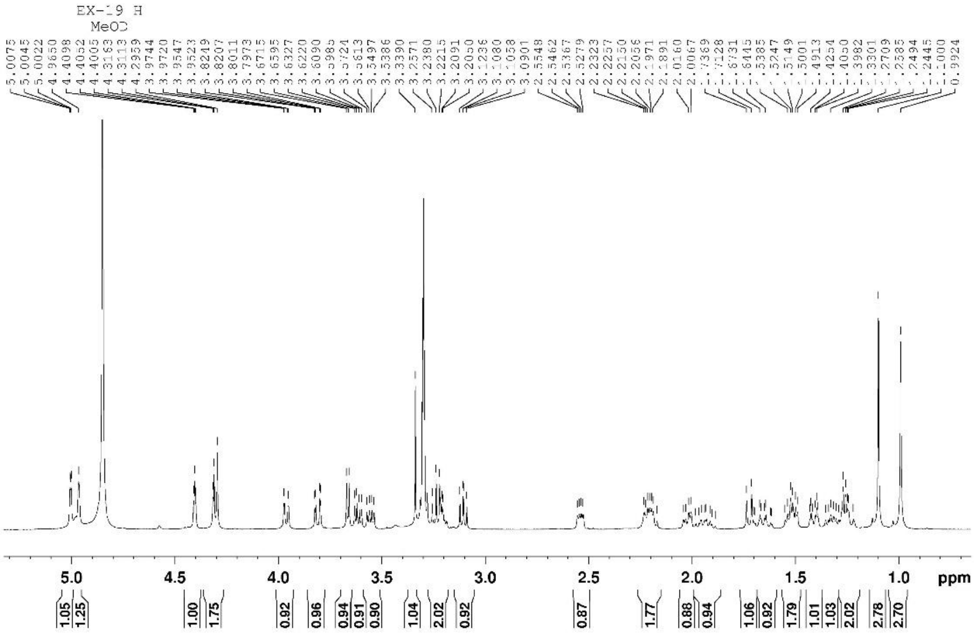

Figure S21. ^{1}H-NMR spectrum of compound **3**.

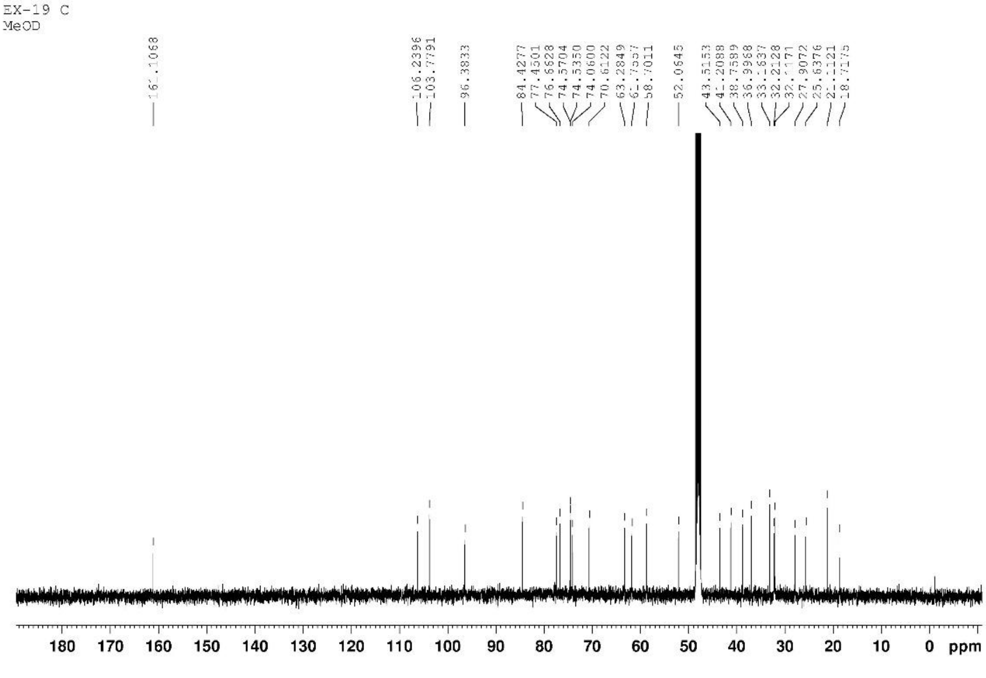

Figure S22. ^{13}C-NMR spectrum of compound **3**.

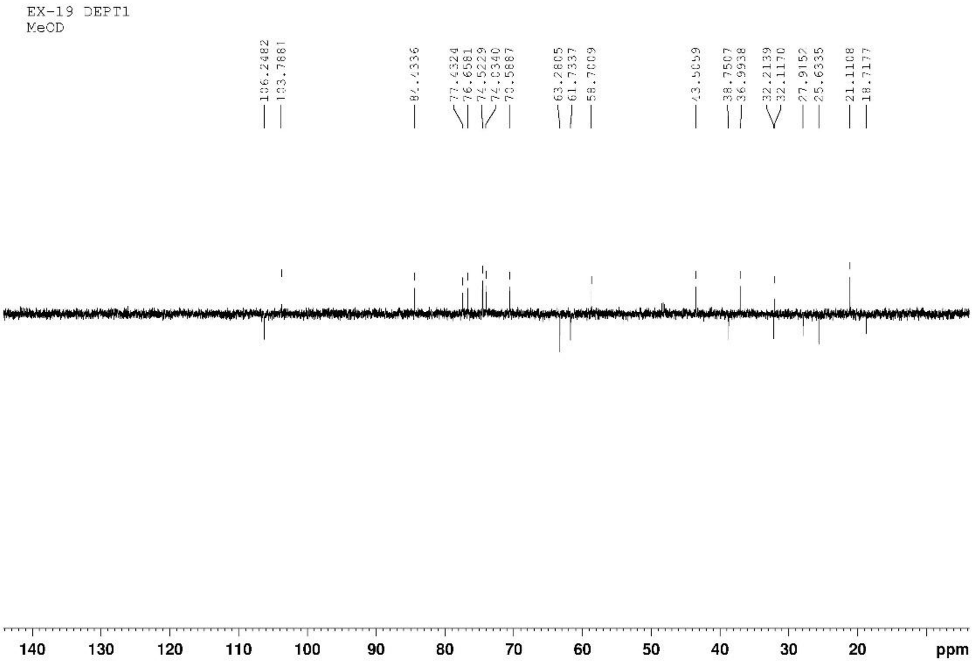

Figure S23. DEPT spectrum of compound **3**.

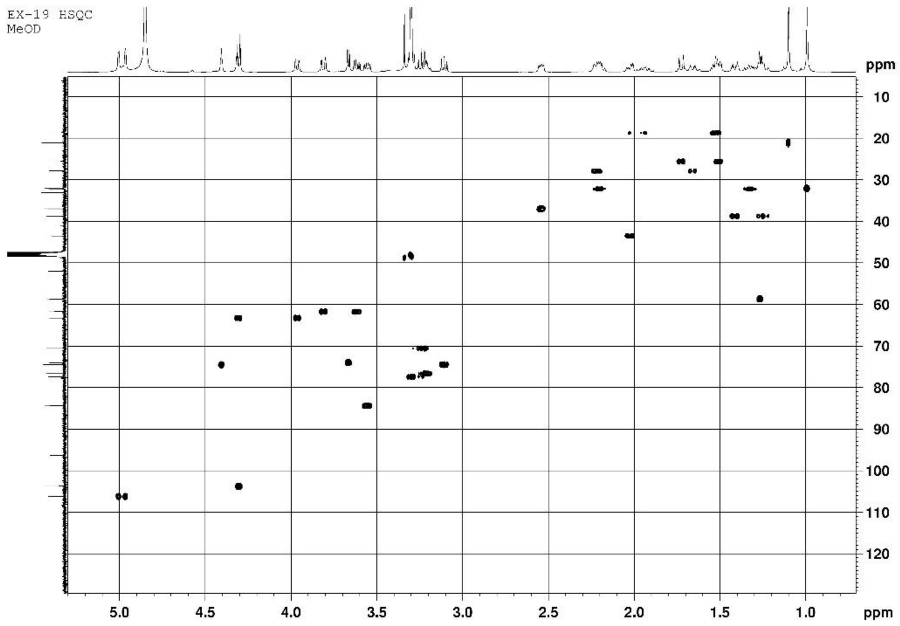

Figure S24. HSQC spectrum of compound **3**.

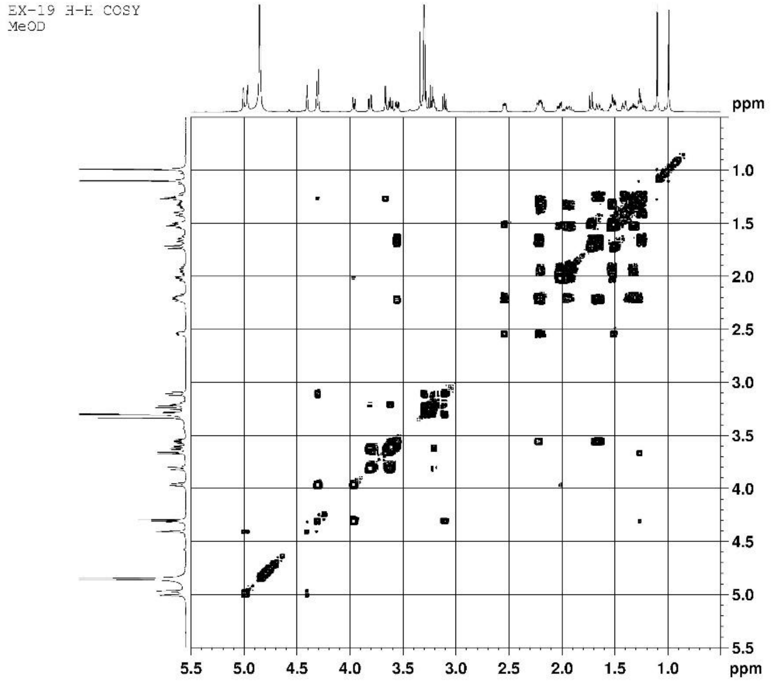

Figure S25. ^{1}H-^{1}H COSY spectrum of compound **3**.

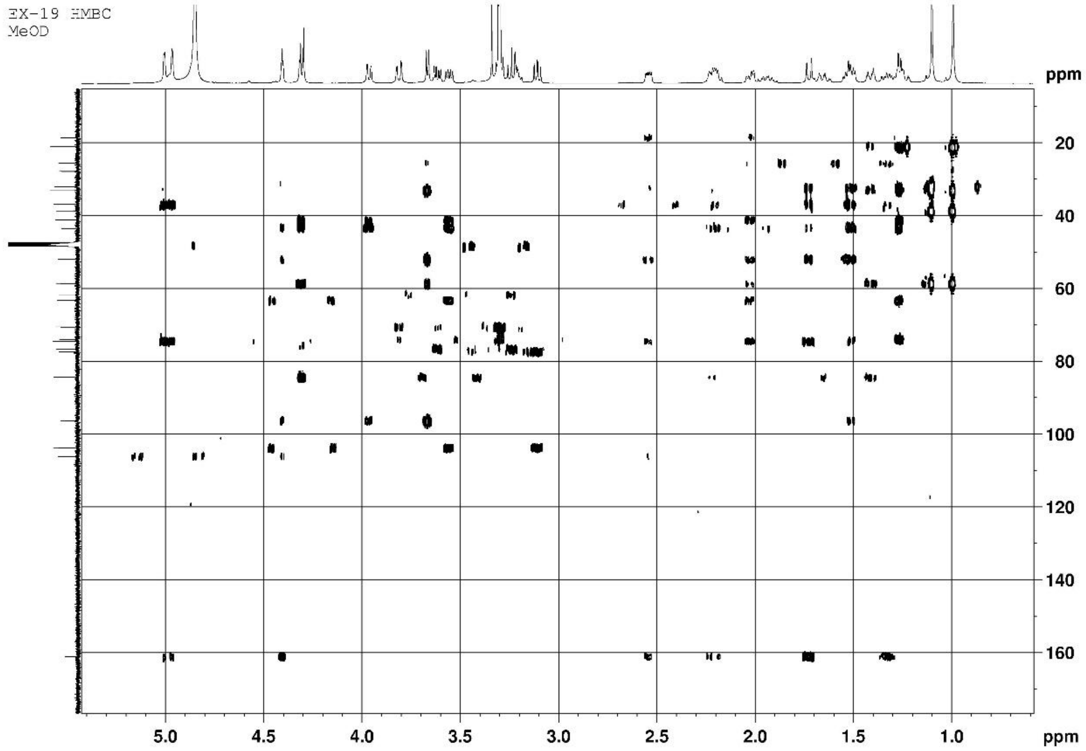

Figure S26. HMBC spectrum of compound **3**.

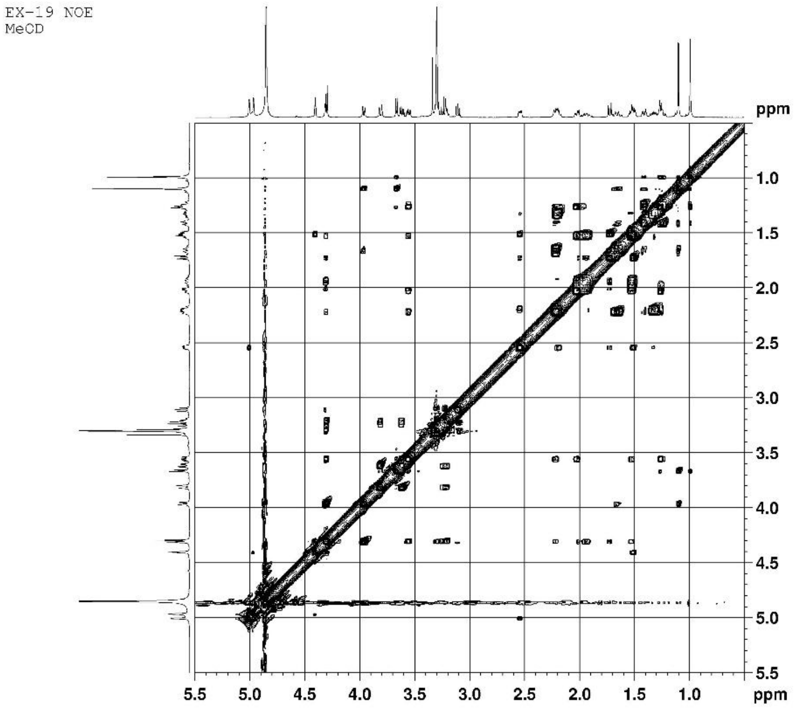

Figure S27. NOESY spectrum of compound **3**.

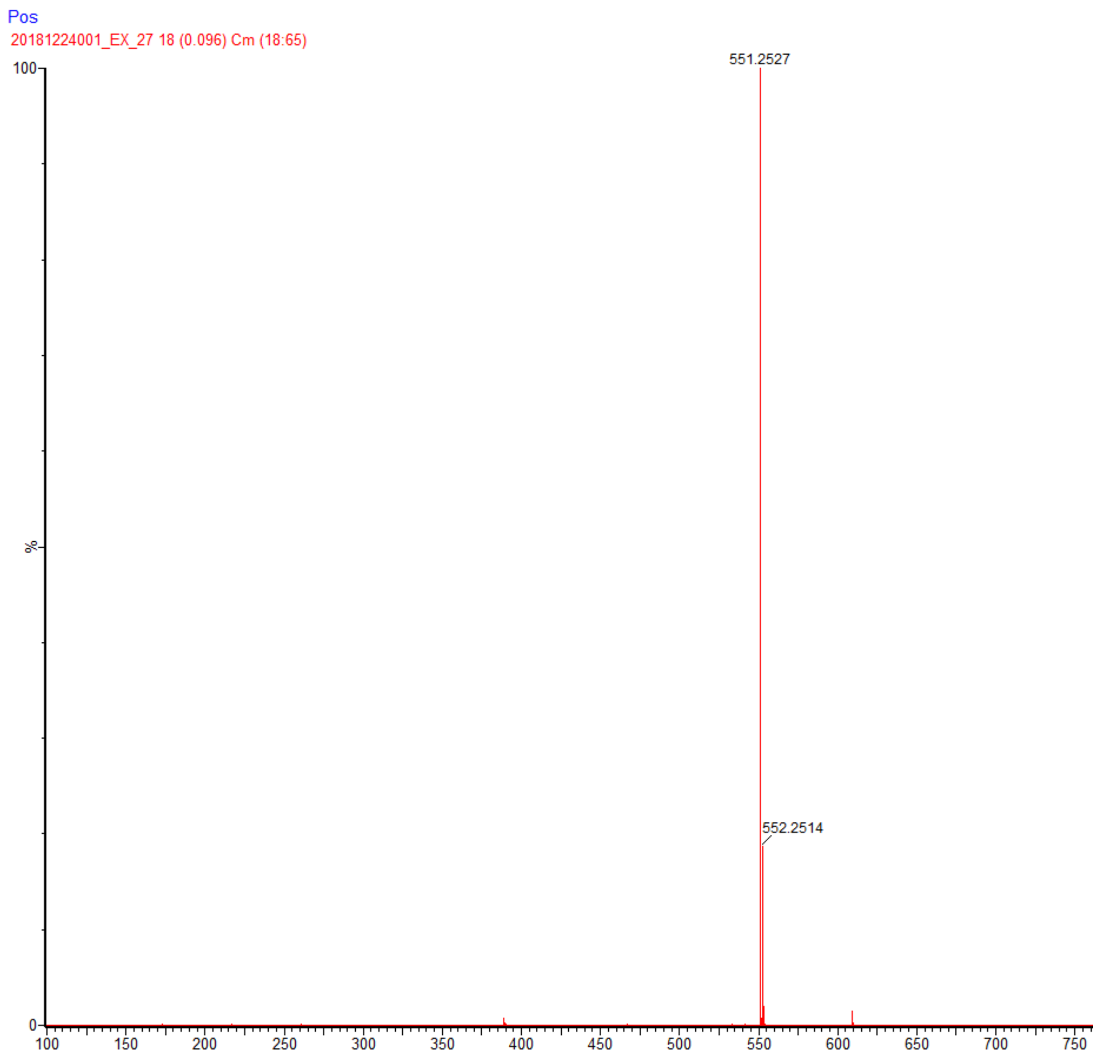

Figure S28. HR-ESI-MS spectrum of compound **4**.

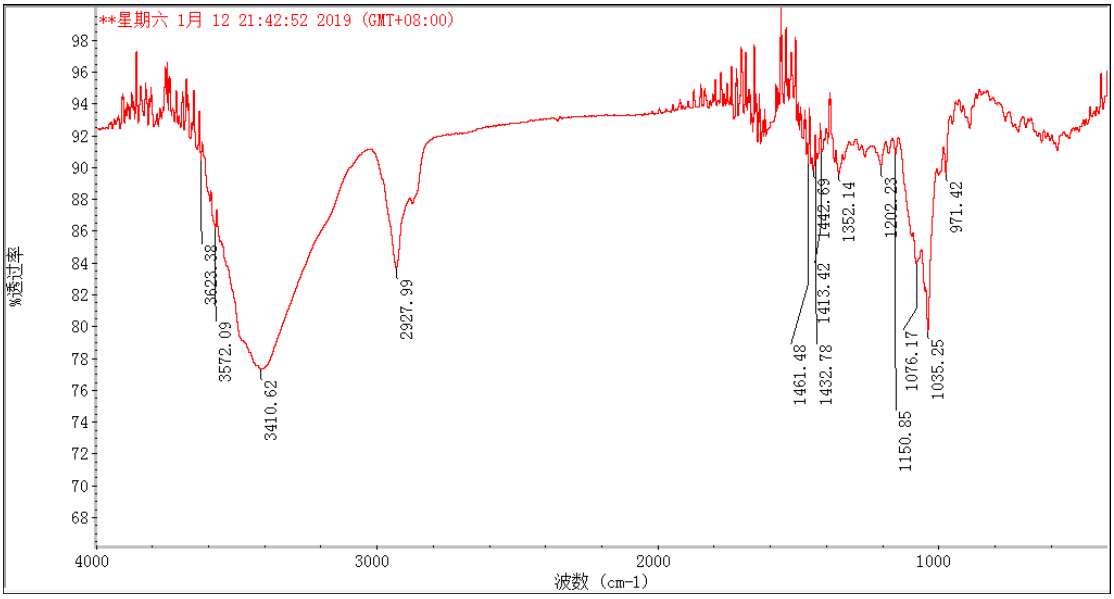

Figure S29. IR spectrum of compound **4**.

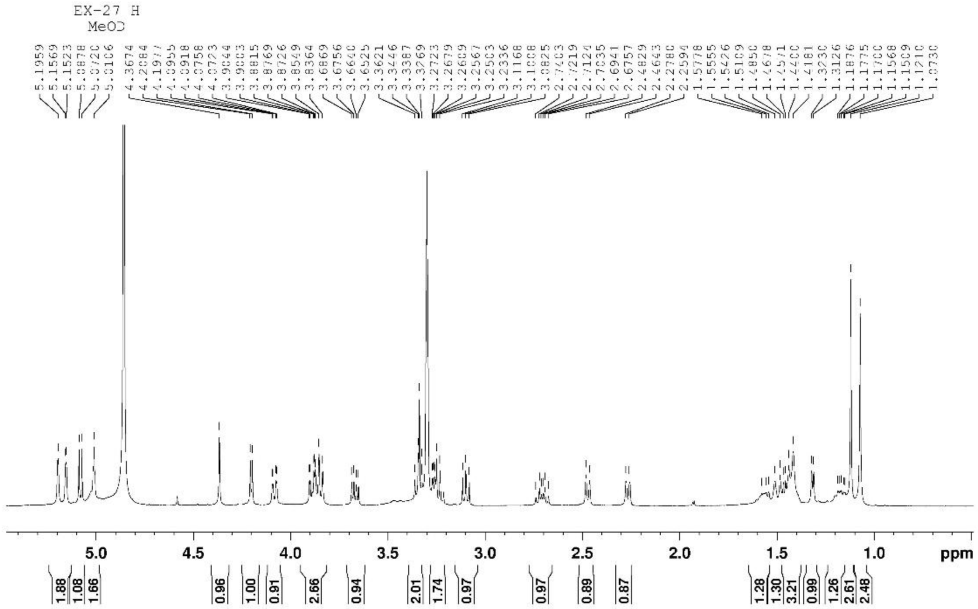

Figure S30. ^{1}H-NMR spectrum of compound **4**.

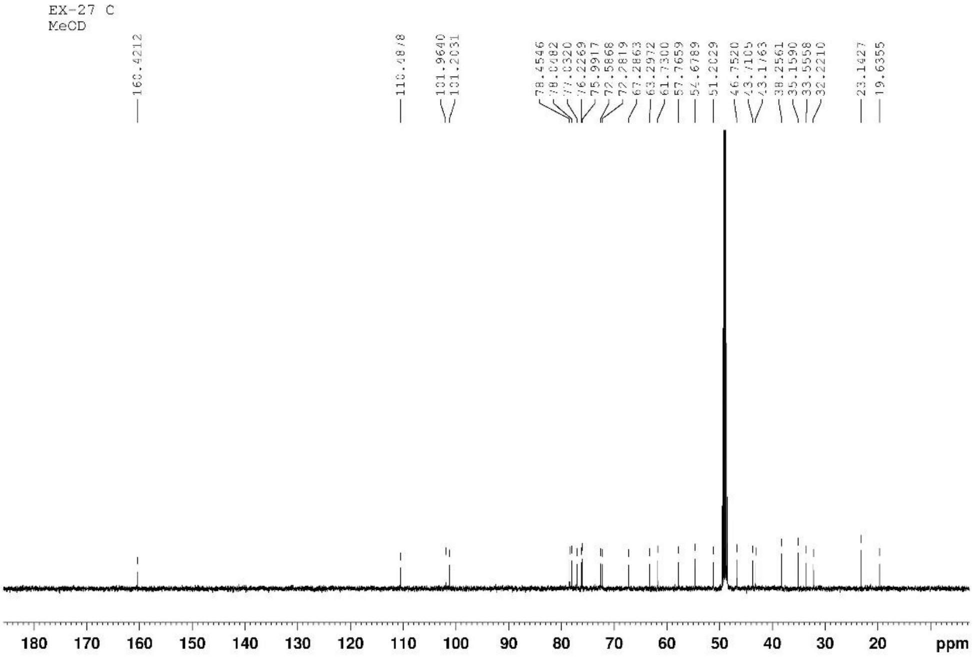

Figure S31. ^{13}C-NMR spectrum of compound **4**.

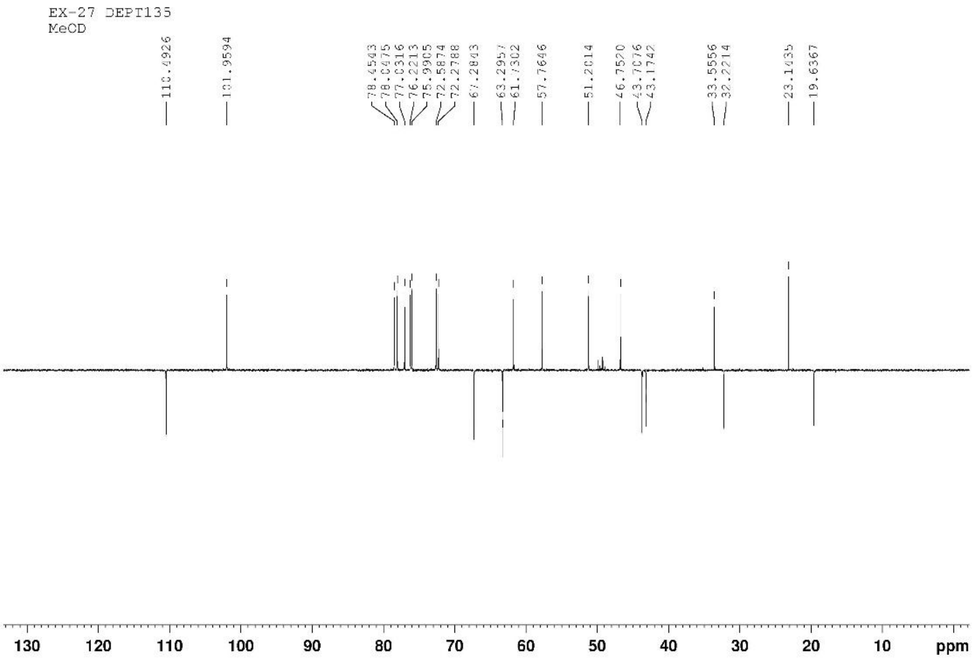

Figure S32. DEPT spectrum of compound **4**.

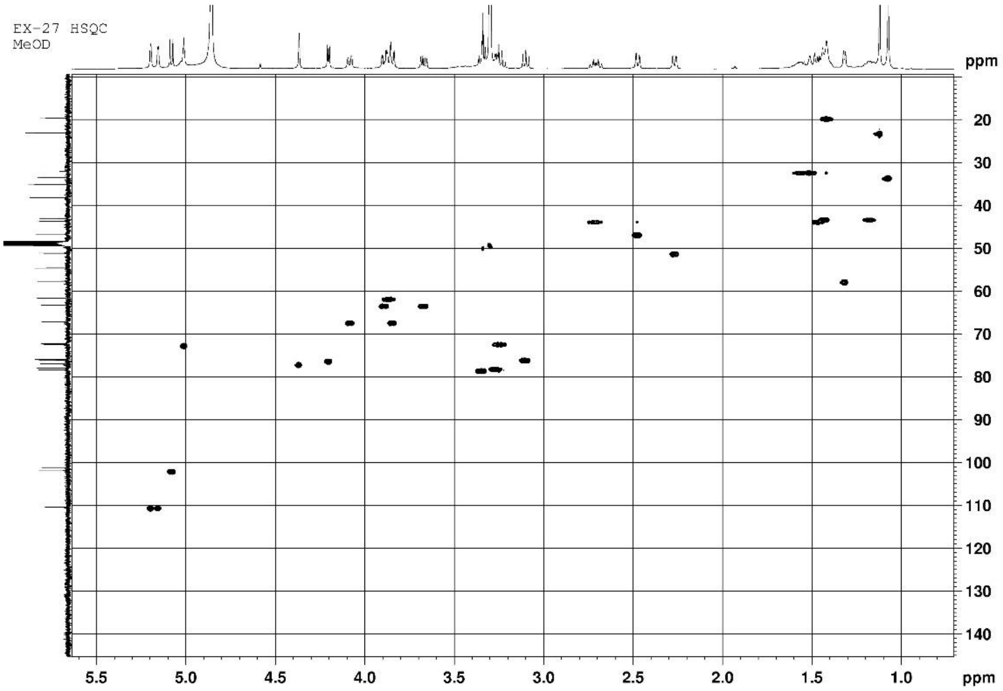

Figure S33. HSQC spectrum of compound **4**.

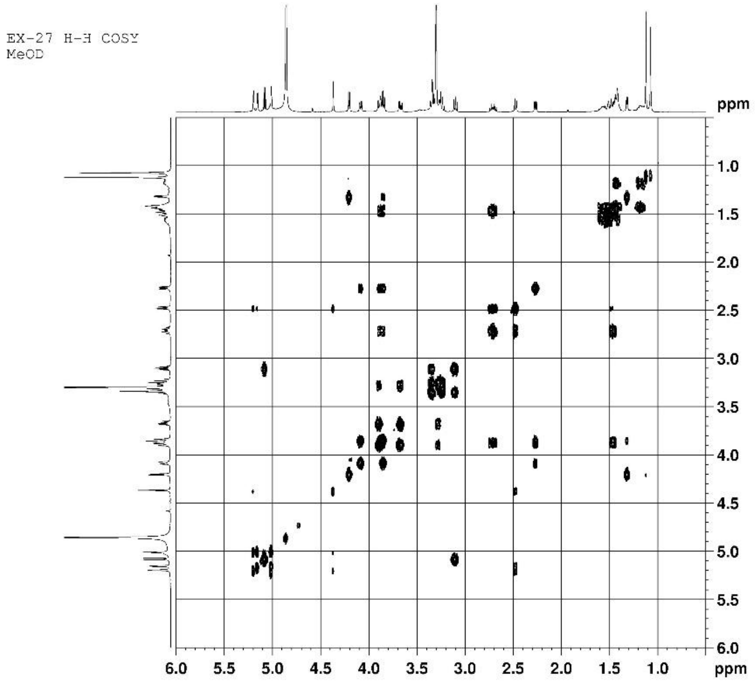

Figure S34. ^{1}H-^{1}H COSY spectrum of compound **4**.

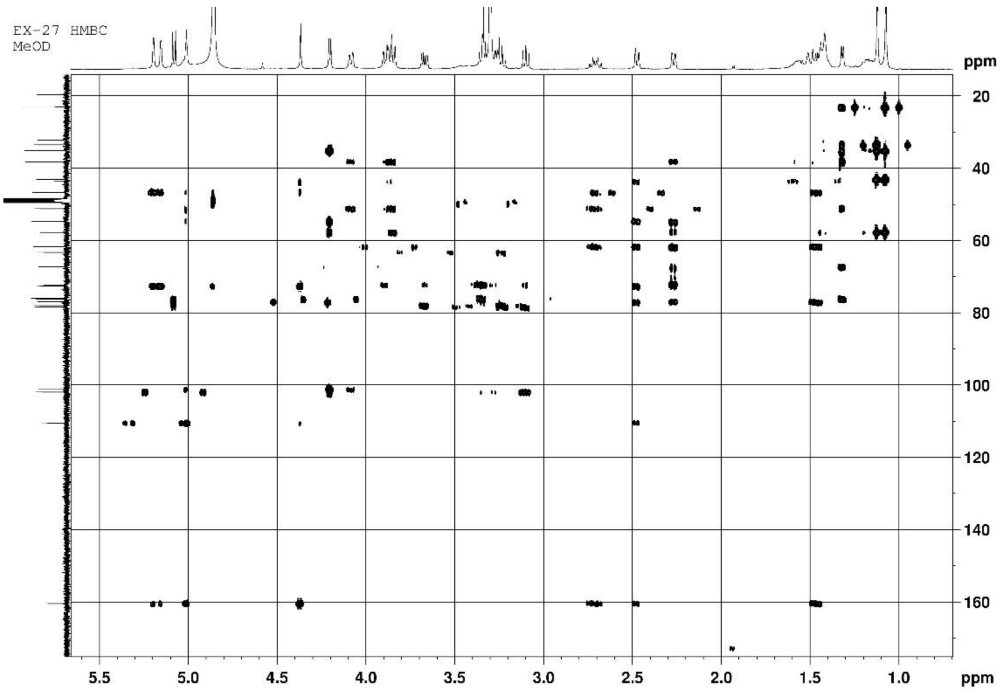

Figure S35. HMBC spectrum of compound **4**.

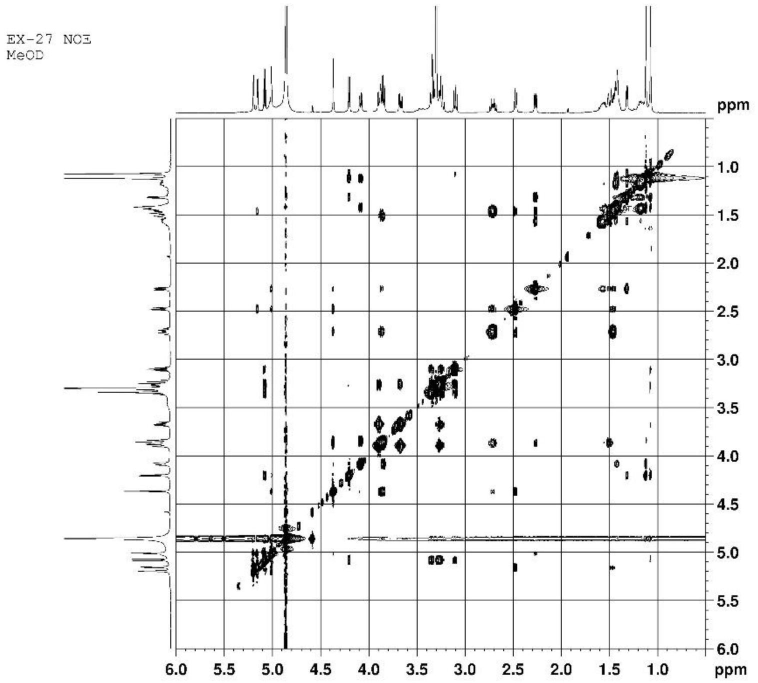

Figure S36. NOESY spectrum of compound **4**.

S37. ECD calculation details of compound of **1**.

1. Methods

Monte Carlo conformational searches were carried out by means of the Spartan's 10 software using Merck Molecular Force Field (MMFF). The conformers with Boltzmann-population of over 2% were chosen for ECD calculations, and then the conformers were initially optimized at B3LYP/6-31+g (d, p) level in MeOH using the CPCM polarizable conductor calculation model. The theoretical calculation of ECD was conducted in MeOH using Time-dependent Density functional theory (TD-DFT) at the B3LYP/6-311+g (d, p) level for all conformers of compounds **EX** . Rotatory strengths for a total of 50 excited states were calculated. ECD spectra were generated using the program SpecDis 1.6 (University of Würzburg, Würzburg, Germany) and GraphPad Prism 5 (University of California San Diego, USA) from dipole-length rotational strengths by applying Gaussian band shapes with sigma = 0.3 eV.

2. Results

Table S1.2.1.Gibbs free energies[a] and equilibrium populations[b] of low-energy conformers of **EX** .

Conformers	In MeOH	
	ΔG	P (%)/100
**EX .-1**	0.00	0.605

**EX .-2**	0.35	0.334
**EX .-3**	1.47	0.051
**EX .-4**	3.07	0.003
**EX .-5**	3.26	0.002

[a]B3LYP/6-31+G(d,p), in kcal/mol. [b]From Δ*G* values at 298.15K.

Table S1.2.2.**Cartesian coordinates for the low-energy reoptimized MMFF conformers of **EX** at B3LYP/6-311+G(d,p) level of theory in CH_3OH.

**EX -1**		Standard Orientation (Ångstroms)			
Center number	Atomic number	Atomic Type	X	Y	Z
1.	6.	0.	-5.030526	1.355006	-0.804108
2.	6.	0.	-4.043595	2.266826	-1.537109
3.	6.	0.	-2.750286	2.543253	-0.731842
4.	6.	0.	-2.120856	1.172249	-0.263416
5.	6.	0.	-3.089461	0.047210	0.228769
6.	6.	0.	-4.383851	-0.012493	-0.598678
7.	6.	0.	-1.050471	1.306788	0.842228
8.	6.	0.	-0.965536	0.004745	1.700079

9.	6.	0.	-0.905988	-1.268346	0.807796
10.	6.	0.	-2.367514	-1.347520	0.217370
11.	6.	0.	-0.688946	-2.658024	1.467092
12.	6.	0.	-0.554105	-3.541890	0.195647
13.	6.	0.	-1.896959	-3.651182	-0.574609
14.	6.	0.	-2.486909	-2.279267	-0.983281
15.	6.	0.	-3.077109	3.535496	0.407680
16.	6.	0.	-1.749274	3.247885	-1.674034
17.	8.	0.	-5.262413	-0.951436	0.033646
18.	6.	0.	-3.350833	0.261623	1.741365
19.	6.	0.	0.307383	-1.287565	-0.194338
20.	6.	0.	0.402148	-2.735592	-0.661614
21.	6.	0.	1.195044	-3.205462	-1.628726
22.	8.	0.	1.511452	-1.051908	0.627913
23.	8.	0.	-2.155917	-0.055605	2.482848
24.	8.	0.	0.192853	1.651537	0.249397
25.	8.	0.	0.078231	0.115753	2.624549
26.	6.	0.	2.635477	-0.462329	0.028653
27.	8.	0.	2.731897	0.871568	0.496740
28.	6.	0.	3.775388	1.622804	-0.160014
29.	6.	0.	5.126027	1.027263	0.273740
30.	6.	0.	5.152177	-0.483269	-0.017515

31.	6.	0.	3.893942	-1.213235	0.474599
32.	8.	0.	3.952785	-2.523133	-0.072668
33.	8.	0.	5.251707	-0.608606	-1.436022
34.	8.	0.	6.215343	1.669684	-0.362564
35.	6.	0.	3.567407	3.085520	0.207175
36.	8.	0.	2.430514	3.645142	-0.420132
37.	8.	0.	-1.839831	-1.679181	-2.110147
38.	1.	0.	-5.944258	1.232137	-1.402424
39.	1.	0.	-5.341082	1.782529	0.156013
40.	1.	0.	-3.775188	1.792186	-2.491386
41.	1.	0.	-4.517727	3.221855	-1.793096
42.	1.	0.	-1.615928	0.773491	-1.149577
43.	1.	0.	-4.107356	-0.382753	-1.598334
44.	1.	0.	-1.336485	2.072542	1.573634
45.	1.	0.	-2.916431	-1.904170	0.983084
46.	1.	0.	0.234808	-2.697654	2.051423
47.	1.	0.	-1.513559	-2.946550	2.122248
48.	1.	0.	-0.178276	-4.547262	0.411383
49.	1.	0.	-1.753205	-4.272472	-1.468633
50.	1.	0.	-2.636264	-4.166752	0.050978
51.	1.	0.	-3.551626	-2.429632	-1.207980
52.	1.	0.	-3.518949	4.441215	-0.022080

53.	1.	0.	-3.789400	3.146176	1.139411
54.	1.	0.	-2.179944	3.847355	0.948734
55.	1.	0.	-2.181448	4.177981	-2.061133
56.	1.	0.	-1.498510	2.613616	-2.531327
57.	1.	0.	-0.817764	3.494025	-1.160045
58.	1.	0.	-6.082840	-0.971601	-0.476275
59.	1.	0.	-4.132267	-0.415782	2.092938
60.	1.	0.	-3.661779	1.286350	1.970238
61.	1.	0.	0.270334	-0.547369	-0.989493
62.	1.	0.	1.812212	-2.554786	-2.241200
63.	1.	0.	1.237783	-4.267505	-1.858147
64.	1.	0.	0.903962	1.430138	0.873824
65.	1.	0.	0.835655	-0.360627	2.246466
66.	1.	0.	2.548142	-0.475628	-1.066540
67.	1.	0.	3.664551	1.520623	-1.248794
68.	1.	0.	5.244715	1.185165	1.352557
69.	1.	0.	6.036316	-0.929695	0.460683
70.	1.	0.	3.885568	-1.234438	1.574835
71.	1.	0.	3.059395	-2.902615	-0.040602
72.	1.	0.	5.092539	-1.545826	-1.626904
73.	1.	0.	6.276656	1.274922	-1.246449
74.	1.	0.	4.437658	3.650562	-0.137268

75.	1.	0.	3.522096	3.170517	1.305983
76.	1.	0.	1.648434	3.126178	-0.170972
77.	1.	0.	-1.770170	-2.339943	-2.811716

**EX -2**		Standard Orientation (Ångstroms)			
Center number	Atom number	Type	X	Y	Z
1.	6.	0.	-5.030222	1.355292	-0.804265
2.	6.	0.	-4.043191	2.266833	-1.537480
3.	6.	0.	-2.749837	2.543242	-0.732293
4.	6.	0.	-2.120593	1.172253	-0.263642
5.	6.	0.	-3.089308	0.047411	0.228767
6.	6.	0.	-4.383735	-0.012262	-0.598632
7.	6.	0.	-1.050177	1.306801	0.841964
8.	6.	0.	-0.965344	0.004852	1.700004
9.	6.	0.	-0.905991	-1.268393	0.807919
10.	6.	0.	-2.367506	-1.347415	0.217489

11.	6.	0.	-0.689212	-2.658005	1.467416
12.	6.	0.	-0.554527	-3.542090	0.196091
13.	6.	0.	-1.897367	-3.651218	-0.574211
14.	6.	0.	-2.486987	-2.279236	-0.983114
15.	6.	0.	-3.076500	3.535665	0.407116
16.	6.	0.	-1.748777	3.247595	-1.674643
17.	8.	0.	-5.262386	-0.951004	0.033878
18.	6.	0.	-3.350610	0.262030	1.741331
19.	6.	0.	0.307388	-1.287968	-0.194230
20.	6.	0.	0.401920	-2.736108	-0.661258
21.	6.	0.	1.194856	-3.206254	-1.628196
22.	8.	0.	1.511487	-1.052332	0.627856
23.	8.	0.	-2.155710	-0.055207	2.482817
24.	8.	0.	0.193170	1.651367	0.249077
25.	8.	0.	0.078426	0.115851	2.624456
26.	6.	0.	2.635378	-0.462524	0.028642
27.	8.	0.	2.731784	0.871220	0.497198
28.	6.	0.	3.774981	1.622832	-0.159589
29.	6.	0.	5.125804	1.027320	0.273646
30.	6.	0.	5.152064	-0.483123	-0.018063
31.	6.	0.	3.894013	-1.213401	0.474101
32.	8.	0.	3.952918	-2.523124	-0.073583

33.	8.	0.	5.251334	-0.608005	-1.436621
34.	8.	0.	6.214853	1.670089	-0.362762
35.	6.	0.	3.566852	3.085405	0.208086
36.	8.	0.	2.429764	3.645094	-0.418738
37.	8.	0.	-1.839635	-1.679388	-2.109961
38.	1.	0.	-5.943982	1.232452	-1.402551
39.	1.	0.	-5.340654	1.783070	0.155785
40.	1.	0.	-3.774873	1.791965	-2.491666
41.	1.	0.	-4.517214	3.221872	-1.793634
42.	1.	0.	-1.615717	0.773249	-1.149720
43.	1.	0.	-4.107296	-0.382734	-1.598229
44.	1.	0.	-1.336103	2.072632	1.573331
45.	1.	0.	-2.916498	-1.903958	0.983228
46.	1.	0.	0.234549	-2.697758	2.051729
47.	1.	0.	-1.513867	-2.946278	2.122629
48.	1.	0.	-0.178915	-4.547512	0.411968
49.	1.	0.	-1.753703	-4.272697	-1.468123
50.	1.	0.	-2.636826	-4.166511	0.051414
51.	1.	0.	-3.551713	-2.429369	-1.207908
52.	1.	0.	-3.518278	4.441371	-0.022732
53.	1.	0.	-3.788773	3.146493	1.138952
54.	1.	0.	-2.179252	3.847485	0.948056

55.	1.	0.	-2.180832	4.177708	-2.061833
56.	1.	0.	-1.498181	2.613169	-2.531868
57.	1.	0.	-0.817189	3.493654	-1.160759
58.	1.	0.	-6.083447	-0.969935	-0.475065
59.	1.	0.	-4.132110	-0.415287	2.092927
60.	1.	0.	-3.661474	1.286812	1.970120
61.	1.	0.	0.270361	-0.547931	-0.989539
62.	1.	0.	1.812175	-2.555767	-2.240718
63.	1.	0.	1.237473	-4.268342	-1.857443
64.	1.	0.	0.904344	1.429635	0.873335
65.	1.	0.	0.835813	-0.360613	2.246425
66.	1.	0.	2.547886	-0.475410	-1.066547
67.	1.	0.	3.663907	1.520923	-1.248372
68.	1.	0.	5.244752	1.184894	1.352488
69.	1.	0.	6.036336	-0.929598	0.459841
70.	1.	0.	3.885886	-1.234943	1.574331
71.	1.	0.	3.059613	-2.902778	-0.041371
72.	1.	0.	5.092302	-1.545184	-1.627786
73.	1.	0.	6.276106	1.275473	-1.246715
74.	1.	0.	4.436973	3.650630	-0.136406
75.	1.	0.	3.521851	3.170061	1.306941
76.	1.	0.	1.647794	3.125749	-0.169943

77.	1.	0.	-1.769503	-2.340398	-2.811246

**EX -3**		Standard Orientation (Ångstroms)			
Center number	Atom number	Type	X	Y	Z
1.	6.	0.	-4.961468	1.364578	-0.941023
2.	6.	0.	-3.932147	2.303468	-1.573471
3.	6.	0.	-2.700453	2.558520	-0.670699
4.	6.	0.	-2.094780	1.174094	-0.211821
5.	6.	0.	-3.087179	0.026468	0.177387
6.	6.	0.	-4.325911	-0.007772	-0.732951
7.	6.	0.	-1.084855	1.275949	0.958801
8.	6.	0.	-1.046891	-0.061285	1.754733
9.	6.	0.	-0.914040	-1.286623	0.816866
10.	6.	0.	-2.354189	-1.365356	0.171161
11.	6.	0.	-0.687303	-2.685508	1.442130
12.	6.	0.	-0.510752	-3.533988	0.153362

13.	6.	0.	-1.833141	-3.646582	-0.650212
14.	6.	0.	-2.428462	-2.274721	-1.050197
15.	6.	0.	-3.114809	3.505400	0.478849
16.	6.	0.	-1.642976	3.303967	-1.514942
17.	8.	0.	-5.244284	-0.959575	-0.180159
18.	6.	0.	-3.448647	0.188212	1.673252
19.	6.	0.	0.323644	-1.249688	-0.161604
20.	6.	0.	0.444724	-2.688318	-0.665033
21.	6.	0.	1.258620	-3.127954	-1.628970
22.	8.	0.	1.502683	-1.001671	0.658534
23.	8.	0.	-2.290266	-0.147372	2.468295
24.	8.	0.	0.184083	1.658291	0.466292
25.	8.	0.	-0.047135	-0.043820	2.732544
26.	6.	0.	2.628570	-0.447128	0.046307
27.	8.	0.	2.726726	0.909571	0.451981
28.	6.	0.	3.767576	1.630832	-0.229858
29.	6.	0.	5.120430	1.055419	0.222964
30.	6.	0.	5.145467	-0.468465	0.014445
31.	6.	0.	3.884919	-1.171103	0.539925
32.	8.	0.	3.949845	-2.510065	0.062976
33.	8.	0.	5.251407	-0.673614	-1.395085
34.	8.	0.	6.206759	1.663983	-0.453276

35.	6.	0.	3.553916	3.102664	0.096654
36.	8.	0.	2.353275	3.602705	-0.457251
37.	8.	0.	-1.766391	-1.643717	-2.151093
38.	1.	0.	-5.830458	1.257344	-1.605268
39.	1.	0.	-5.342323	1.759258	0.008016
40.	1.	0.	-3.594528	1.861385	-2.521296
41.	1.	0.	-4.393236	3.264473	-1.830623
42.	1.	0.	-1.533522	0.809429	-1.077926
43.	1.	0.	-3.985316	-0.353085	-1.720993
44.	1.	0.	-1.433405	2.013829	1.696745
45.	1.	0.	-2.925417	-1.941698	0.905627
46.	1.	0.	0.225473	-2.703398	2.042509
47.	1.	0.	-1.520186	-3.008552	2.071700
48.	1.	0.	-0.120729	-4.537679	0.351416
49.	1.	0.	-1.662213	-4.250901	-1.551214
50.	1.	0.	-2.582926	-4.179895	-0.052246
51.	1.	0.	-3.485763	-2.433421	-1.302898
52.	1.	0.	-3.533834	4.423653	0.052873
53.	1.	0.	-3.873676	3.084496	1.143536
54.	1.	0.	-2.259849	3.803567	1.091574
55.	1.	0.	-2.057859	4.243044	-1.899157
56.	1.	0.	-1.326481	2.702457	-2.373862

57.	1.	0.	-0.751536	3.541293	-0.930860
58.	1.	0.	-6.021681	-0.980559	-0.753511
59.	1.	0.	-4.241657	-0.507954	1.954371
60.	1.	0.	-3.778127	1.202064	1.921818
61.	1.	0.	0.261458	-0.505416	-0.952709
62.	1.	0.	1.877552	-2.458861	-2.218934
63.	1.	0.	1.321944	-4.185538	-1.874578
64.	1.	0.	0.878677	1.286546	1.036987
65.	1.	0.	-0.403810	0.494749	3.455561
66.	1.	0.	2.564071	-0.502591	-1.050409
67.	1.	0.	3.656765	1.493263	-1.315406
68.	1.	0.	5.246318	1.269126	1.291458
69.	1.	0.	6.028487	-0.888199	0.518779
70.	1.	0.	3.865590	-1.135042	1.638822
71.	1.	0.	3.049938	-2.873567	0.084639
72.	1.	0.	5.067363	-1.616279	-1.530609
73.	1.	0.	6.264940	1.217329	-1.312282
74.	1.	0.	4.379265	3.677727	-0.331771
75.	1.	0.	3.588220	3.227963	1.192480
76.	1.	0.	1.621022	3.047206	-0.141381
77.	1.	0.	-1.611355	-2.305776	-2.837382

**EX -4**		Standard Orientation (Ångstroms)			
Center number	Atom number	Type	X	Y	Z
1.	6.	0.	-5.381248	-0.518577	0.923898
2.	6.	0.	-4.604746	-1.536810	1.760917
3.	6.	0.	-3.422491	-2.183872	0.999039
4.	6.	0.	-2.511832	-1.055933	0.371409
5.	6.	0.	-3.218959	0.213914	-0.231708
6.	6.	0.	-4.456609	0.643995	0.573216
7.	6.	0.	-1.544924	-1.581839	-0.735073
8.	6.	0.	-1.151930	-0.385563	-1.654376
9.	6.	0.	-0.796380	0.897552	-0.853018
10.	6.	0.	-2.201548	1.405367	-0.369999
11.	6.	0.	-0.169370	2.072501	-1.637119
12.	6.	0.	0.145479	3.040670	-0.472205
13.	6.	0.	-1.150870	3.594715	0.176493
14.	6.	0.	-2.117893	2.499719	0.693329
15.	6.	0.	-3.982945	-3.202575	-0.020075

16.	6.	0.	-2.578338	-2.979439	2.020466
17.	8.	0.	-5.122871	1.670013	-0.175255
18.	6.	0.	-3.545451	-0.086305	-1.714857
19.	6.	0.	0.278095	0.714243	0.290970
20.	6.	0.	0.841019	2.115214	0.507686
21.	6.	0.	1.758025	2.474166	1.407234
22.	8.	0.	1.236982	-0.278179	-0.180781
23.	8.	0.	-2.308547	-0.108953	-2.461846
24.	8.	0.	-0.472336	-2.359623	-0.252221
25.	8.	0.	-0.138844	-0.707939	-2.572780
26.	6.	0.	2.617514	-0.096566	-0.201617
27.	8.	0.	3.159400	-0.484252	1.054489
28.	6.	0.	4.575131	-0.234628	1.119202
29.	6.	0.	5.306035	-1.105067	0.080066
30.	6.	0.	4.699455	-0.921542	-1.318715
31.	6.	0.	3.170307	-1.004016	-1.304028
32.	8.	0.	2.721425	-0.583918	-2.579327
33.	8.	0.	5.109529	0.370541	-1.767328
34.	8.	0.	6.695200	-0.821263	0.061754
35.	6.	0.	4.987660	-0.536220	2.554789
36.	8.	0.	4.220701	0.213752	3.484063
37.	8.	0.	-1.730133	1.932446	1.950101

38.	1.	0.	-6.236645	-0.134209	1.496786
39.	1.	0.	-5.793047	-0.971197	0.014426
40.	1.	0.	-4.217287	-1.027871	2.654450
41.	1.	0.	-5.271842	-2.327684	2.124377
42.	1.	0.	-1.897872	-0.698755	1.203475
43.	1.	0.	-4.090875	1.059954	1.524232
44.	1.	0.	-2.105638	-2.247565	-1.400884
45.	1.	0.	-2.586509	1.967751	-1.226865
46.	1.	0.	0.754687	1.766532	-2.137994
47.	1.	0.	-0.843794	2.482474	-2.393221
48.	1.	0.	0.783661	3.878306	-0.771972
49.	1.	0.	-0.885706	4.267090	1.003475
50.	1.	0.	-1.690745	4.204159	-0.559325
51.	1.	0.	-3.113576	2.953127	0.794528
52.	1.	0.	-4.625873	-3.918059	0.504183
53.	1.	0.	-4.587159	-2.747100	-0.809703
54.	1.	0.	-3.186462	-3.780597	-0.496039
55.	1.	0.	-3.184862	-3.764989	2.486387
56.	1.	0.	-2.211173	-2.325411	2.819655
57.	1.	0.	-1.712418	-3.440566	1.541456
58.	1.	0.	-5.893873	1.948355	0.336214
59.	1.	0.	-4.158341	0.709124	-2.143156

60.	1.	0.	-4.073125	-1.036401	-1.849073
61.	1.	0.	-0.134955	0.321438	1.217620
62.	1.	0.	2.231628	1.766774	2.079707
63.	1.	0.	2.094708	3.505942	1.473838
64.	1.	0.	0.286899	-1.772944	-0.081087
65.	1.	0.	-0.500217	-1.393693	-3.153640
66.	1.	0.	2.878086	0.946066	-0.412344
67.	1.	0.	4.766444	0.826586	0.901424
68.	1.	0.	5.195743	-2.156656	0.373004
69.	1.	0.	5.098498	-1.694008	-1.992569
70.	1.	0.	2.859814	-2.035744	-1.078052
71.	1.	0.	1.744337	-0.595928	-2.567503
72.	1.	0.	4.559418	0.565476	-2.541900
73.	1.	0.	6.794826	-0.042357	-0.507910
74.	1.	0.	6.036264	-0.263951	2.692832
75.	1.	0.	4.890185	-1.618405	2.738598
76.	1.	0.	3.299829	-0.027178	3.309842
77.	1.	0.	-1.496196	2.652504	2.550228

EX -5		Standard Orientation (Ångstroms)			
Center number	Atom number	Type	X	Y	Z
1.	6.	0.	-4.913731	1.543811	-0.578593
2.	6.	0.	-3.883438	2.544656	-1.105027
3.	6.	0.	-2.609629	2.632390	-0.228970
4.	6.	0.	-2.035113	1.182471	0.021536
5.	6.	0.	-3.046927	0.002864	0.239771
6.	6.	0.	-4.309044	0.143269	-0.628535
7.	6.	0.	-1.067064	1.086158	1.216410
8.	6.	0.	-1.001259	-0.375418	1.758879
9.	6.	0.	-0.904344	-1.429694	0.617131
10.	6.	0.	-2.344196	-1.375300	-0.022679
11.	6.	0.	-0.703432	-2.926134	0.980212
12.	6.	0.	-0.520539	-3.526632	-0.440012
13.	6.	0.	-1.843823	-3.475803	-1.250168
14.	6.	0.	-2.430440	-2.048492	-1.388618
15.	6.	0.	-2.942211	3.419271	1.059365
16.	6.	0.	-1.558507	3.451825	-1.010918
17.	8.	0.	-5.231976	-0.874879	-0.222376
18.	6.	0.	-3.373785	-0.090239	1.754993

19.	6.	0.	0.331488	-1.241510	-0.335400
20.	6.	0.	0.445277	-2.553898	-1.091977
21.	6.	0.	1.230531	-2.799075	-2.143124
22.	8.	0.	1.501221	-1.152610	0.564374
23.	8.	0.	-2.221236	-0.595915	2.462766
24.	8.	0.	0.210711	1.599009	0.872234
25.	8.	0.	0.017351	-0.466301	2.718813
26.	6.	0.	2.704511	-0.699268	-0.006570
27.	8.	0.	2.488534	0.597462	-0.502918
28.	6.	0.	3.628619	1.279871	-1.042222
29.	6.	0.	4.744490	1.371743	0.015563
30.	6.	0.	5.029771	-0.001709	0.633562
31.	6.	0.	3.750721	-0.689577	1.111127
32.	8.	0.	4.113209	-2.010918	1.495182
33.	8.	0.	5.653282	-0.781351	-0.388253
34.	8.	0.	5.927374	1.915558	-0.545026
35.	6.	0.	3.088573	2.651059	-1.455588
36.	8.	0.	2.415403	3.294492	-0.391455
37.	8.	0.	-1.770844	-1.230410	-2.359616
38.	1.	0.	-5.815745	1.567198	-1.206047
39.	1.	0.	-5.234396	1.789227	0.440237
40.	1.	0.	-3.598281	2.243504	-2.122788

41.	1.	0.	-4.324509	3.544388	-1.194117
42.	1.	0.	-1.471280	0.934995	-0.883406
43.	1.	0.	-4.002263	-0.032294	-1.671260
44.	1.	0.	-1.459630	1.649953	2.071586
45.	1.	0.	-2.923151	-2.063757	0.600596
46.	1.	0.	0.199121	-3.088730	1.576741
47.	1.	0.	-1.548154	-3.343141	1.532727
48.	1.	0.	-0.143098	-4.554393	-0.425149
49.	1.	0.	-1.675892	-3.903058	-2.247845
50.	1.	0.	-2.598277	-4.105724	-0.762232
51.	1.	0.	-3.490007	-2.153479	-1.660041
52.	1.	0.	-3.365291	4.393176	0.789173
53.	1.	0.	-3.671181	2.917656	1.702150
54.	1.	0.	-2.047220	3.614700	1.655858
55.	1.	0.	-1.943935	4.454370	-1.230256
56.	1.	0.	-1.319087	2.972082	-1.966598
57.	1.	0.	-0.627915	3.561967	-0.450242
58.	1.	0.	-6.024534	-0.782840	-0.767172
59.	1.	0.	-4.184583	-0.801365	1.924335
60.	1.	0.	-3.673386	0.874794	2.178855
61.	1.	0.	0.319381	-0.352479	-0.958213
62.	1.	0.	1.829398	-2.024203	-2.611545

63.	1.	0.	1.286811	-3.790146	-2.587340
64.	1.	0.	0.798568	1.345207	1.599439
65.	1.	0.	0.778970	-0.847649	2.250900
66.	1.	0.	3.030163	-1.375944	-0.810283
67.	1.	0.	4.016451	0.743551	-1.921938
68.	1.	0.	4.407360	2.062489	0.795548
69.	1.	0.	5.714176	0.115709	1.486447
70.	1.	0.	3.332221	-0.124617	1.958753
71.	1.	0.	3.294935	-2.514216	1.609229
72.	1.	0.	5.700353	-1.686534	-0.046347
73.	1.	0.	6.364884	1.180928	-1.002652
74.	1.	0.	2.432393	2.521944	-2.330297
75.	1.	0.	3.929869	3.285837	-1.748057
76.	1.	0.	1.668310	2.725269	-0.138680
77.	1.	0.	-1.584782	-1.767306	-3.140762

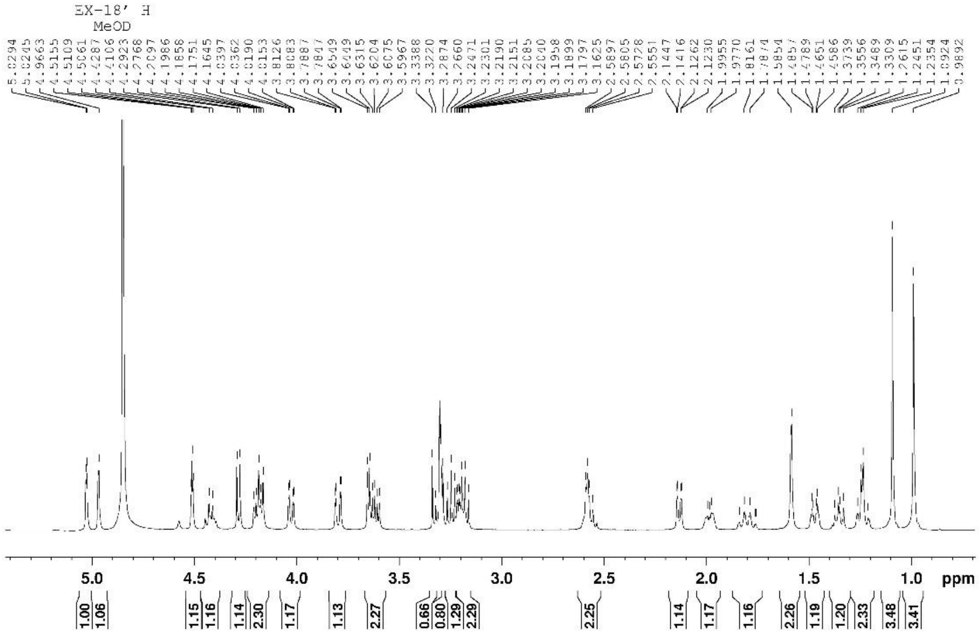

Figure S38. ¹H-NMR spectrum of compound **5**.

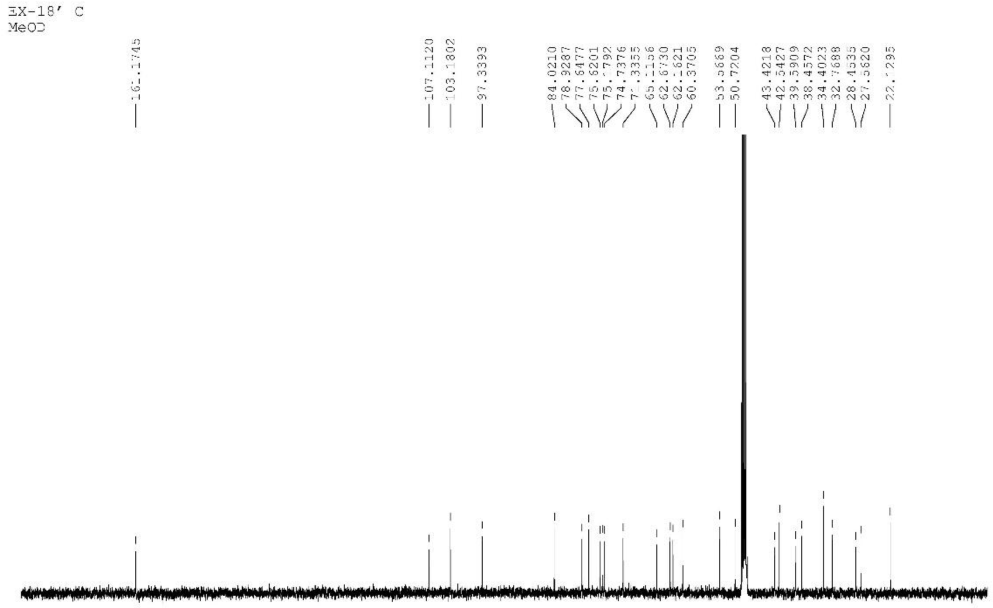

Figure S39. ¹³C-NMR spectrum of compound **5**.

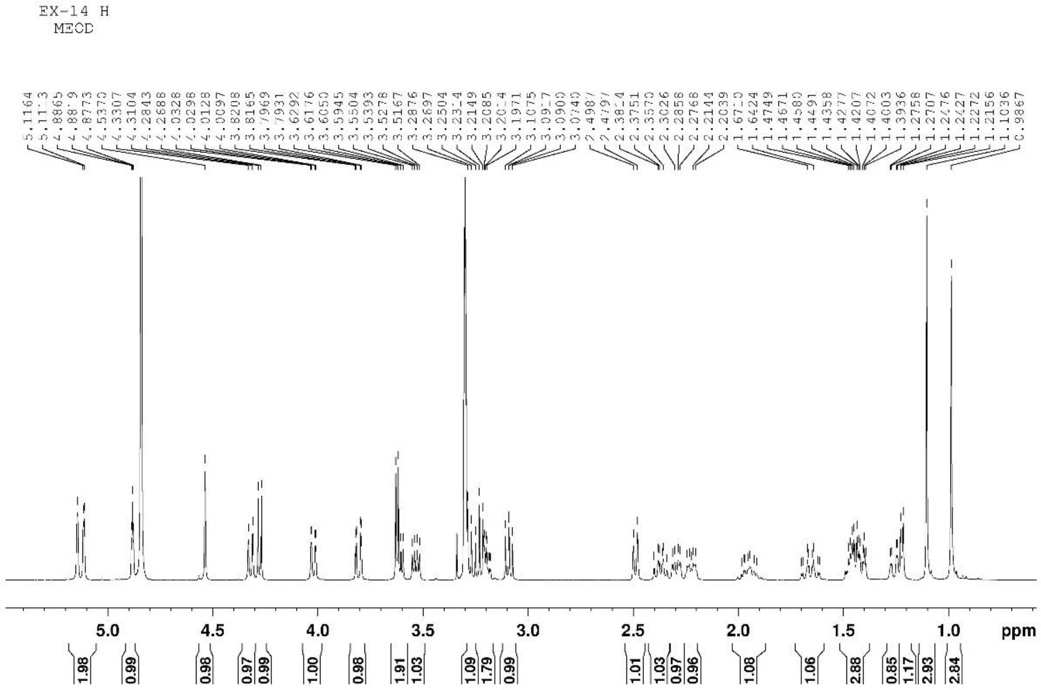

Figure S40. ^{1}H-NMR spectrum of compound **6**.

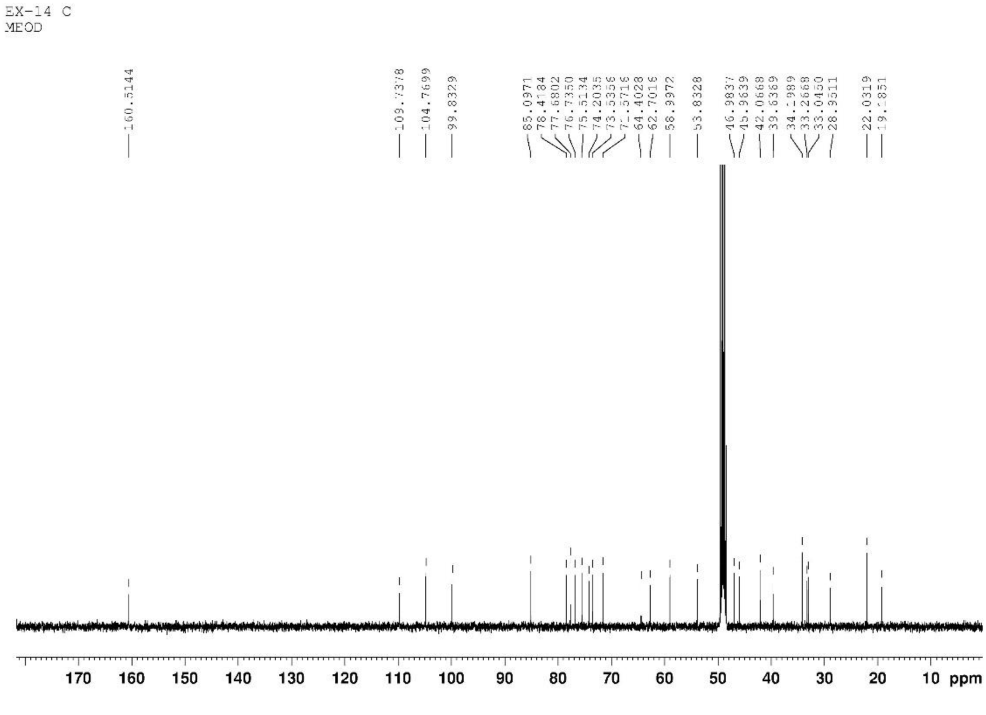

Figure S41. ^{13}C-NMR spectrum of compound **6**.

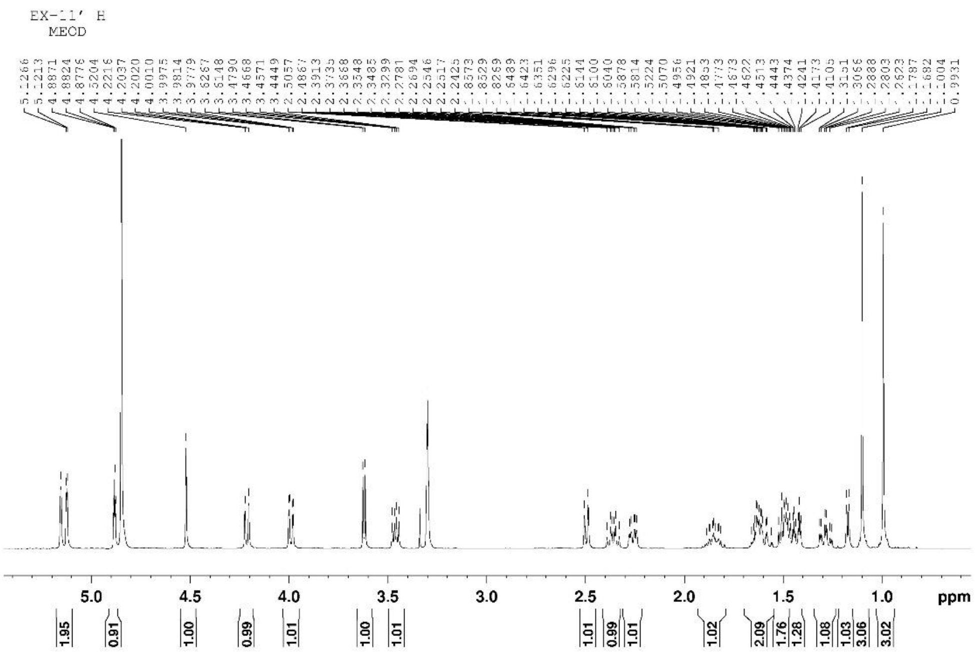

Figure S42. ^{1}H-NMR spectrum of compound **7**.

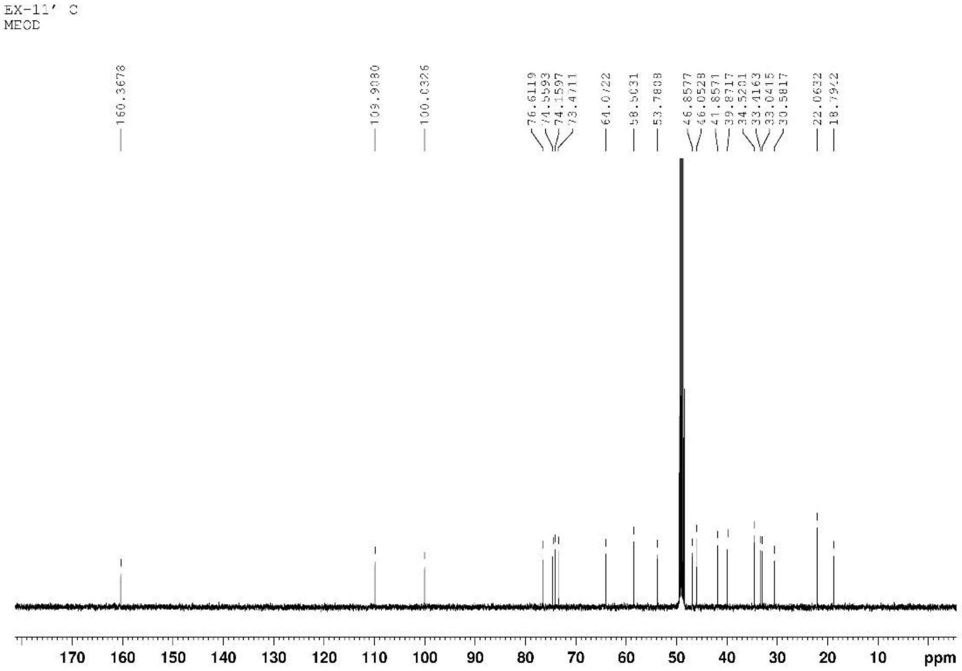

Figure S43. ^{13}C-NMR spectrum of compound **7**.
